# LncRNAOmics: A Comprehensive Review of Long Non-Coding RNAs in Plants

**DOI:** 10.3390/genes16070765

**Published:** 2025-06-29

**Authors:** Chinmay Saha, Saibal Saha, Nitai P. Bhattacharyya

**Affiliations:** 1 Department of Biology, University of North Carolina at Chapel Hill, Chapel Hill, NC 27599, USA; 2 Howard Hughes Medical Institute, University of North Carolina at Chapel Hill, Chapel Hill, NC 27599, USA; 3 Department of Botany, University of Kalyani, Kalyani, West Bengal 741235, India; bt24resch11012@iith.ac.in; 4 Formerly at Crystallography and Molecular Biology Division, Saha Institute of Nuclear Physics, Kolkata, West Bengal 700064, India

**Keywords:** non-coding RNA, long non-coding RNA, microRNA, abiotic stress, biotic stress, epigenetics, LncRNA-interacting proteins, immune response, plant growth and development

## Abstract

The large portion of the eukaryotic genomes was considered non-functional and called the “dark matter” of the genome, now appearing as regulatory hubs coding for RNAs without the potential for making proteins, known as non-coding RNA. Long non-coding RNA (lncRNA) is defined as functional RNA molecules having lengths larger than 200 nucleotides without the potential for coding for proteins. Thousands of lncRNAs are identified in different plants and animals. LncRNAs are characterized by a low abundance, fewer exons than mRNA, tissue-specific expression, and low sequence conservation compared to protein-coding genes (PCGs). LncRNAs, like PCGs, are regulated by promoters and enhancers with characteristic chromatin signatures, DNA methylation, multiple exons, introns, and alternate splicing. LncRNAs interact with DNA, mRNA, microRNA, and proteins, including chromatin/histone modifiers, transcription factors/repressors, epigenetic regulators, spliceosomal, and RNA-binding proteins. Recent observations indicate that lncRNAs code for small peptides, also called micropeptides (<100 amino acids), and are involved in the development and growth of plants, suggesting the bi-functional activities of lncRNAs. LncRNAs have emerged as the major regulators of diverse functions, principally by altering the transcription of target genes. LncRNAs are involved in plant growth, development, immune responses, and various physiological processes. Abiotic, biotic, nutrient, and other environmental stresses alter the expressions of numerous lncRNAs. Understanding the mechanisms of actions of lncRNAs opens up the possibility of improving agronomic traits by manipulating lncRNAs. However, further studies are required in order to find the interactions among the deregulated lncRNAs and validate the findings from high-throughput studies to harness their potential in crop improvement.

## 1. Introduction

Sequencing thousands of eukaryotic genomes, including plant genomes, and the prediction of protein-coding genes revealed that large parts of the genomes do not code for proteins. In last 20 years, the sequencing of the number of plant genomes completely or partially increased considerably. In 2000, the genome sequence of *Arabidopsis* was first reported. Presently, the sequences of 4604 plant genomes from 1482 plant species have been published. The genome sizes of plants vary from a few million to a few billion base pairs (bp). For example, the genome sizes of *Arabidopsis*, *Oryza sativa* (rice), *Zea mays* (Maize), *Hordeum vulgare* (Barley), and *Triticum aestivum* (wheat) are about 135 Mb, 500 MB, 2.2 billion bp, 5.3 billion bp, and 17 billion bp, respectively [1]. However, genome sizes, determined by the amount of DNA or the nucleotide numbers obtained by the genome sequencing of the eukaryotic organisms, and computationally predicted or experimentally determined number of protein-coding genes (PCGs) by RNA sequencing, do not significantly correlate with the developmental complexity of organisms [2,3]. The vast majority of the genome that does not code for a protein-coding gene (PCG) is commonly known as “non-coding DNA,” also called as “junk DNA,” due to the presence of simple repetitive sequences, transposons, and pseudogenes [4]. The results of many studies using high-throughput sequencing revealed that about 90% of the eukaryotic genome is transcribed; approximately 2% of the genome is translated into proteins [4,5,6]. The portions of the genomes are transcribed and code for RNAs that do not code for any functional proteins and are sometimes regarded as the “dark matter” of the genomes [7,8,9].

In light of recent advances, we adopt the term LncRNAOmics to describe the growing field, focusing on the comprehensive studies of plant long non-coding RNAs (lncRNAs). LncRNAOmics brings together high-throughput sequencing, advanced computational analysis, and integrative biological approaches to explore the full landscape of plant lncRNAs. This emerging area of research covers everything from the discovery of lncRNAs to their classification, expression patterns, molecular interactions, and functional roles under various developmental stages and biotic and abiotic stresses. By taking a holistic view, LncRNAOmics offers valuable insights into how lncRNAs contribute to plant growth, the overall regulation of gene expression, and stress responses. In the present review, we attempt to provide an overview of the up-to-date state of knowledge in this exciting and rapidly evolving field.

## 2. Non-Coding RNA Coded by Plant Genomes

**Figure 1:**
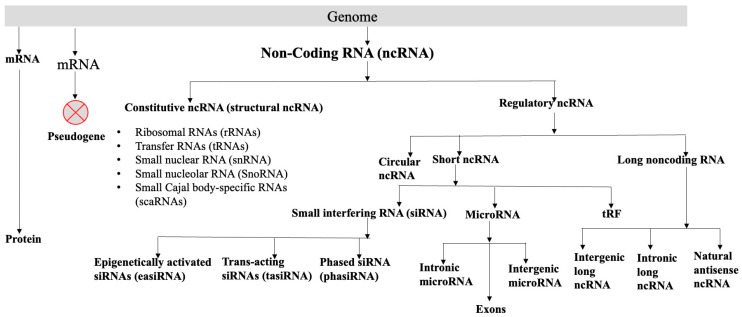
Classification of different types of RNAs.

A major portion of the genome codes for RNAs, which are not translated into proteins. A non-coding RNA (ncRNA) is a functional RNA molecule, without the potential to code for a protein. The plant genomes, like animal genomes, code for many types of RNAs like well-characterized ribosomal RNAs (rRNAs) and transfer RNAs (tRNAs), all involved in protein synthesis. Small nuclear RNAs (snRNAs), and small nucleolar RNAs (SnoRNAs) participate in splicing. MicroRNAs (miRNAs) are negative regulators of PCGs. Circular RNAs (circRNAs), small interfering RNAs (siRNAs), tRNA-derived small RNA fragments (tRFs), and long non-coding RNAs (lncRNAs) are involved directly or indirectly in the regulation of gene expression. These RNAs can broadly be divided into two categories, constitutive ncRNA and regulatory ncRNA (Figure 1, Box 1), although they have overlap in their roles. The snRNA, snoRNA, and scaRNA might also regulate different biological processes [10]. Regulatory ncRNAs can be divided into (a) circular RNAs, (b) short ncRNAs, and (c) long ncRNAs. Short RNA consists of siRNA, microRNA, and tRF. Small interfering RNAs can be further classified into (i) phased siRNA (phasiRNA), (ii) trans-acting siRNAs (tasiRNA), and (iii) epigenetically activated siRNAs (easiRNA). The functions and the biogenesis of different classes of siRNAs are reviewed [11]. MicroRNAs are endogenous, short, non-coding RNAs that are 20–24 nucleotides long and generally negatively regulate the expression of the target genes (mRNAs) post-transcriptionally and play important roles in plant development and growth, and biotic and abiotic stresses. They code from intergenic regions, exons, or intronic regions of the genomes [12,13,14]. Many plant species code for circular RNAs, which are non-coding RNAs. Because of their distinctive closed-loop architectures, circular RNAs are a special class of endogenous ncRNAs. The non-canonical “back-splicing” process, in which a covalent bond joins the 5′ and 3′ ends, creates circular RNAs. Numerous eukaryotes, such as humans, animals, and plants, code circular RNAs [15] (https://plant.deepbiology.cn/PlantCircRNA/, accessed on 2 March 2025). The tRFs, which are produced by cleaving tRNA at different positions and usually consist of 17–26 nucleotides, are crucial for plant development, growth, stress reactions, and other biological functions [16]. Single-stranded RNA molecules longer than 200 nucleotides that are functional but do not have the ability to code for proteins are known as long non-coding RNAs (lncRNAs). Long non-coding RNA (lncRNA) is defined as a functional single-stranded RNA molecule > 200 nucleotides long without having the potential for coding protein. Depending on the genomic regions from where the lncRNAs are coded, they can be classified as promoter-associated lncRNA [17], natural antisense transcripts [18,19], intronic lncRNA [20], and intergenic lncRNA [21,22]. Even though lncRNA coded by the enhancer is known in animals [23], thus far, no conclusive evidence for the presence of enhancer-coded lncRNAs in plants is available, possibly due to the non-availability of well-characterized enhancers in plants [24]. For details of the different classes of ncRNAs, their biogenesis, and possible functions, see the current reviews [25,26,27,28], briefly summarized in Figure 1 and Box 1.

Box 1 **Different types of non-coding RNAs.**

 Constitutive ncRNA (structural ncRNA)

 Ribosomal RNA: [18S (~1800 nucleotides), 5.8S (~161 nucleotides), and 25S (3376 nucleotides) rRNA gene coded by 45S rDNA; 5S rRNA (~121 nucleotides) is coded by 5S rDNA] [29,30].

 Transfer RNA (tRNA): All organisms code transfer RNAs (tRNAs), which are non-coding RNAs of an intermediate size (73–91 nucleotides) that are involved in the translation of messenger RNA into protein [31].

 Small nuclear RNA (snRNA): snRNAs are non-coding RNAs found mainly in the nucleus of a cell. They are essential for processing pre-messenger RNAs (pre-mRNAs), particularly in splicing, where they are vital components of the spliceosome. In addition to splicing, snRNAs also contribute to RNA maturation and gene expression regulation [32]. While telomerase RNA (TR) shares some structural features with snRNAs, it functions as a distinct non-coding RNA in plants, acting as a template for synthesizing telomeric repeats essential for chromosome end maintenance [33].

 Small nucleolar RNA (snoRNA): SnoRNAs are short, non-coding RNA molecules that play a key role in ribosome formation by directing the chemical changes that rRNA undergoes. They are crucial for rRNA synthesis, protein assembly, folding, and modifications (such as 2′O-methylation and pseudouridylation), all of which are necessary for the development of ribosomes. Arabidopsis has been found to contain SnoRNAs [34].

 Small Cajal body-specific RNAs (scaRNAs): scaRNAs are a type of small nucleolar RNA (snoRNA) that modify spliceosomal RNAs in plant cells. These modifications occur in the Cajal body, a nuclear organelle that helps create small nuclear ribonucleoproteins [10].

 Regulatory ncRNA

 tRNA-derived small RNA fragments (tRFs): tRFs, which usually consist of 17–26 nucleotides, are produced by cleaving tRNA at different locations.

 Small interfering RNAs (siRNAs): Plant defense and stress responses are two mechanisms in which the short ncRNAs, siRNAs (20–25 bp), are crucial. Dicer-like enzymes can detect hairpin-like structures formed by miniature inverted-repeat transposable element (MITE) transcripts and break them into short RNAs, particularly siRNAs. This process has been confirmed in rice and Arabidopsis [27].

 MicroRNA: A class of endogenous, short, non-coding RNAs with a length of 20–24 nucleotides, microRNAs (miRNAs) regulate the expression of their target genes (mRNAs) post-transcriptionally and play a role for the normal growth, development, and response to both abiotic and biotic stressors in plants [12,13,14].

 Circular RNA: Circular RNAs (circRNAs) are non-coding RNAs coded by many plant species. Because of their distinctive closed-loop topologies, circular RNAs (circRNAs) constitute a special class of endogenous non-coding RNAs (ncRNAs). The non-canonical “back-splicing” process, in which a covalent bond joins the 5′ and 3′ ends, creates circular RNAs. They are found in many eukaryotes, including plants, animals, and humans (https://plant.deepbiology.cn/PlantCircRNA/, accessed on 2 March 2025).

 Long non-coding RNA (lncRNA): Typically, longer than 200 nucleotides, lncRNAs are functional heterogeneous RNA molecules that do not have the ability to translate into proteins. They are involved in a number of important molecular and biological processes, including organogenesis in roots, photomorphogenesis in seedlings, abiotic stress responses, silence of genes, flowering time regulation, and reproduction [24].

 Pseudogene: Plant pseudogenes are non-functional copies of protein-coding genes. Pseudogenes are genomic fossils that are formed by duplication, retrotransposition, or disabling mutations [35].

Since long non-coding RNA is the focus of this review, we will go into further detail about various aspects of the lncRNA. However, we quickly go over the different components of microRNA, which commonly negatively regulate the expression of genes that code for proteins. MicroRNAs interacting with lncRNAs may indirectly modulate their target protein-coding genes. To understand the mechanisms of microRNA–lncRNA interactions, we briefly describe below the biogenesis and mechanisms of action of microRNA on the target protein-coding genes.

### 2.1. Biogenesis of MicroRNA and Mode of Action in Plants

Thousands of microRNAs in eukaryotes, such as plants and animals, have been discovered as a result of the advancement in high-throughput sequencing technology and analytic tools. Numerous databases, such as miRBase (miRBase https://www.mirbase.org/, accessed on 2 March 2025 [36]), catalogues and present the microRNAs observed in plants and other organisms. There are other databases dedicated to plant microRNAs. For instance, 8433 miRNAs from 121 plant species are listed in the plant miRNA database (PMRD, http://bioinformatics.cau.edu.cn/PMRD/, accessed on 2 March 2025 [37]), which includes model plants Arabidopsis and important crops such as rice, wheat, soybean, maize, sorghum, barley, and others. The targets of miRNA, secondary structures, expression patterns, and sequence information of the miRNAs are also included in this database. Another comprehensive functional plant miRNA database is Plant miRNA ENcyclopedia (PmiREN). Version 2.0 of this database catalogues 141,327 predicted miRNA-target pairs in 179 plant species and 38,186 miRNA loci (https://pmiren.com/, accessed on 2 March 2025 [38]). A plant miRNA-target collection called TarDB (http://www.biosequencing.cn/TarDB/, accessed on 2 March 2025 [39]) has 62,888 cross-species conserved miRNA targets from 43 different plant species. The representative number of microRNAs coded by the genomes of plants is shown in Table 1. MicroRNA synthesized within cells of a particular tissue can affect not only the targets within that specific cell but also tissues at a distance [40,41], or sometimes in other organisms [41,42,43]. However, the exact mechanisms of cell–cell or organism–organism interactions remain largely unknown. MicroRNA targeting protein-coding genes perform diverse biological functions, including development, reproduction, and the response to external conditions [44,45,46], details of which are beyond the scope of the present review.

The production of mature microRNA in plants occurs in multiple stages: (i) the transcription of the microRNA gene into the primary transcript of miRNA (pri-miRNA), followed by 3′-polyadenylation and 5′-capping; (ii) the cleavage of the pri-miRNA to miRNA-miRNA* duplex by Dicer-like RNase III endonucleases (DCLs) in collaboration with the zinc-finger protein Serrate (SE) and the double-stranded RNA-binding protein Hyponastic Leaves 1 (HYL1); (iii) the methylation of the 2′-OH position by RNA methyltransferase HUA Enhancer 1 (HEN1); (iv) the export of the methylated miRNA–miRNA* duplex to cytoplasm via Hasty (HST), a plant homolog of the animal Exportin 5 (EXPO5); (v) the assembly of RISC, and the separation and degradation of the passenger strand (miRNA*), and loading of the driver strand (mature, functional miRNA) to the RISC-AGO1 complex; and (vi) the interaction with the target mRNA and mRNA. The details of the biogenesis and mechanism of actions of microRNA are described in Supplementary Text S1.

### 2.2. Long Non-Coding RNA in Plants

Functional transcripts longer than 200 nucleotides that do not have the potential to code for proteins are known as long non-coding RNAs (lncRNAs). Hundreds of thousands of lncRNAs that are encoded by the genomes of hundreds of plants and animals have been discovered, as a result of the development of high-throughput DNA and RNA sequencing technologies and efficient bioinformatics tools. Thousands of plant species have been found to contain lncRNAs. For instance, Arabidopsis and agricultural plants such as cotton, wheat, rice, maize, and numerous other species have been found to have lncRNAs [1]. Plant genomes code many non-coding RNAs, including lncRNAs, and can be obtained from different repository databases (see the following section for the representative result). LncRNAs vary considerably across species, ranging from around 2000 in a single-cell green alga *Chlamydomonas reinhardtii* to over 23,000 in *Z. mays* [47]. Table 2 shows the representative data for lncRNAs coded by the plant genomes. For comparison, we also included the genomes of humans and mice. Compared to mRNAs, lncRNAs are less abundant, contain fewer exons, and are less evolutionarily conserved across species, according to a global survey of lncRNAs from mammals [48,49]. The majority of plant lncRNAs identified and confirmed by low-throughput assays do not exhibit cross-species conservation. As a result, lncRNAs are a family of molecules that are changing quickly and might not need conserved domains to work like proteins. In order to control the chromatin structure, RNA activity, or protein changes, the expressed lncRNAs often attach to DNA, RNAs, or proteins. In order to control the chromatin structures, gene expression, RNA activity, or protein changes, the expressed lncRNAs interact with DNA, RNAs, or proteins [50].

#### Biogenesis of Long Non-Coding RNA

Loci expressing lncRNAs (lncRNA genes) show many of the characteristics of protein-coding genes, including promoters and enhancers with the characteristic chromatin signatures, DNA methylation, multiple exons, introns, alternate splicing, regulation by conventional transcription factors, and altered expression in response to external stress or genetic makeup. RNA polymerase I (pol I) primarily transcribes rRNAs, while RNA pol III transcribes tRNA, snRNA, and other types of RNA. Similar to mRNA, RNA Pol II transcribes the majority of lncRNAs from the sense or antisense strand. Plant-specific RNA Pol V transcribes a small percentage of lncRNAs. Several lncRNAs, including APOLO, ASCO, COLDAIR, COLDWRAP, COOLAIR, SVALKA, and many more, are transcribed by Pol II. Plant RNA Pol IV transcribes short non-coding RNA, while Pol V transcribes small numbers of lncRNAs. Pol IV- and Pol V-produced transcripts seem to be involved in RNA-directed DNA methylation (RdDM) and transposable element silencing [29]. Like protein-coding genes, lncRNAs have introns and exons, although lncRNAs generally possess limited exons and introns. For example, rice lncRNAs have fewer exons than mRNAs; the average number of exons in lncRNA was 2.21 in comparison to 4.67 exons in mRNAs. Compared to protein-coding genes (median length of 159 nucleotides), lncRNAs have longer average exon lengths (median length of 323 nucleotides) [52]. The spliceosome, a large non-coding RNA–protein complex made up of core small nuclear ribonucleoproteins, U-rich major and minor short nuclear RNAs, and a number of auxiliary RNA-binding proteins, splices lncRNAs just like it does mRNAs. LncRNAs are primarily polyadenylated at the 3′ end (poly A tail) and protected by 5′-capping. Some lncRNAs, on the other hand, do not have poly A tails. In response to abiotic stress in Arabidopsis, hundreds of lncRNAs that are not polyadenylated are identified. These lncRNAs are characterized by the significant instability and reduced expression levels [24]. In animal-cell-based experimental settings, the half-lives of lncRNAs range from 30 min to several hours, with an average half-life of 4.8 h. In contrast, the mean half-life of protein-coding genes was 7.7 h. This result shows that lncRNAs are not generally unstable, although their half-lives are shorter than mRNA [53]. Similar to mammalian cell culture, Arabidopsis’ mRNA stability varies from a few minutes to several hours under normal circumstances, although it may change in response to stress [54]. The rapid turnover of lncRNAs is initiated by decapping, deadenylation, and also exonucleolytic digestion due to their interactions with partners like microRNA.

### 2.3. Classification of lncRNA Based on Their Chromosomal Locations and Direction of Transcription

**Figure 2:**
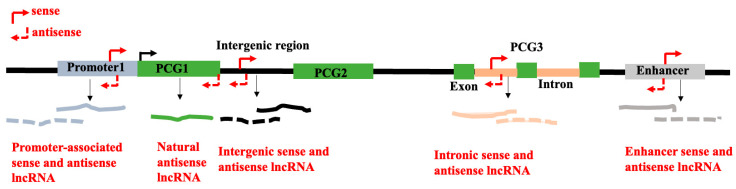
Long non-coding RNA (lncRNA) is coded by the genome (thick black horizontal line). Depending on the genomic regions from where the lncRNA is coded, they are classified as promoter-associated lncRNA, enhancer-associated lncRNA, genic lncRNA, intronic lncRNA, and intergenic lncRNA, and shown by different colors. Depending on the direction of the transcription of the lncRNAs (indicated by red arrows; sense—solid arrow; antisense—dashed arrow) concerning the protein-coding genes (PCGs), lncRNAs are designated as sense (solid line) or antisense (dashed line) lncRNAs. Examples of different types of lncRNAs are promoter-associated lncRNA in Arabidopsis COLDWRAP [17], natural antisense lncRNA asDOG1 in Arabidopsis [18], intergenic LDMAR in hybrid rice [21], and intronic lncRNA in rice RIFLA [20]. Even though lncRNA coded by the enhancer is known in animals [23], no report is available on plants [24].

There is no consensus classification scheme for lncRNAs. Different classifications of lncRNAs have been proposed based on their length, subcellular localization, genomic positions, and patterns of transcription with respect to the protein-coding genes, and activities [55]. The most widely utilized characteristic for classifying lncRNAs is their genomic positions in relation to known genomic annotations of the protein-coding genes. Depending on the genomic locations of the lncRNAs and their direction of transcription with respect to the protein-coding genes, they can be classified into promoter-associated lncRNAs, genic lncRNAs, intronic lncRNAs, and intergenic lncRNAs. Depending on the direction of the transcription of the lncRNAs, lncRNAs are designated as sense or antisense lncRNAs (Figure 2). For examples of these types of lncRNAs in plants, please see the legends of Figure 2. Based on the mechanism of actions of lncRNAs as detailed below, they also can be classified into (a) decoy/sponge lncRNAs, (b) scaffold lncRNAs, (c) guide lncRNAs, and (d) signalling lncRNAs (for details, see Section 4). Such classification systems highlight the regulatory mechanisms of action of lncRNAs. Moreover, lncRNAs can be generically classified as either trans-acting or cis-acting. While trans-acting lncRNAs exit the transcription sites and carry out biological tasks in trans, cis-acting lncRNAs control the chromatin structure and expression of neighboring genes in cis. Some short RNAs like miRNAs, short hairpin RNAs (shRNAs), and siRNAs may reside within the genomic regions coding for lncRNAs [56,57].

### 2.4. Resources for Plant lncRNAs

There are many databases cataloging hundreds of thousands of lncRNAs from different plant species. Table 3 shows the representative databases for plant lncRNAs. We mention a few of them only. A comprehensive functional plant lncRNA database is the Plant Long Non-coding RNA Database (PLncDB). In the current version (v2.2, 10 August 2021), PLncDB contains 1,246,372 lncRNAs from 80 species ranging from chlorophytes to embryophytes [58]. A total of 571,688 lncRNAs from 108 plant species have been computationally identified from high-throughput transcriptome sequencing data and catalogued in the CANTATAdb 3.0 database. Of the lncRNAs, 112,980 were expressed only in specific plant organs or embryos, suggesting possible functions in organ-specific processes and development [59]. The Green Non-Coding Database (GreeNC, v2.0) is a plant and algae long non-coding lncRNAs database. Presently (v2.0), it describes the annotation of more than 495,000 annotated lncRNAs from 94 species [60]. Database PlantNATsDB [61] contains information on the natural antisense lncRNAs from different plant species. The plant ncRNA database (PNRD) is a repository of plant lncRNA [62]. It was found that 203,391 known and predicted lncRNA sequences from nine species—*Z. mays* L., *Gossypium barbadense* L., *Triticum aestivum* L., *Lycopersicon esculentum* Mille, *O. sativa* L., *Hordeum vulgare* L., *Sorghum bicolor* L., *Glycine max* L., and *Cucumis sativus* L.—are included in the functional database LncPheDB. A genome-wide association analysis was used to catalog the link between 68,862 lncRNAs and the genomic location of variations. Various agronomic traits were linked to genetic variations [63].

### 2.5. Micropeptide Coded by the lncRNA in Plants

**Figure 3:**
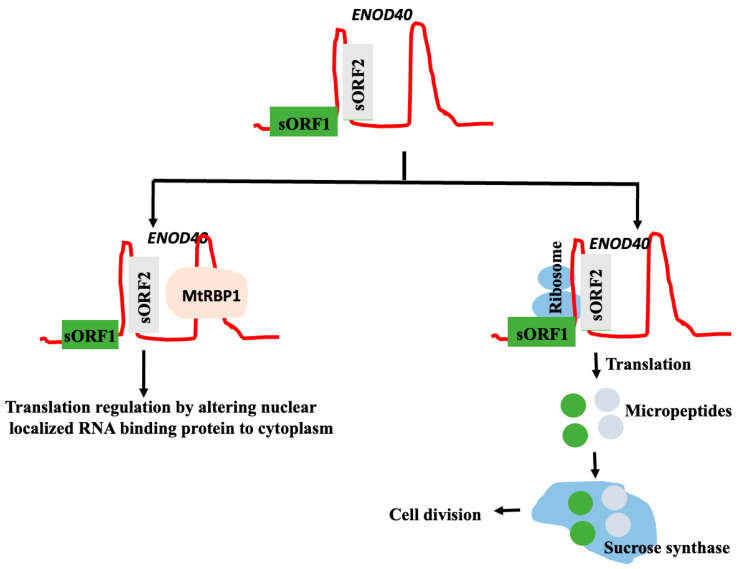
Bi-functional activity of LncRNA ENOD40. The lncRNA is about 700 nt long, identified in many plants like *Medicago truncatula*, *Oryza sativa*, *Arabidopsis thaliana*, *Glycine max* (soya beans); codes for two micropeptides; and is conserved more in the regions outside the peptide-coding regions [68]. Two micropeptides coded by the ENOD40 interact with sucrose synthase and regulates cell division in soya beans [69]. ENOD4 directly interacts with *M. truncatula* RNA-binding protein 1 (MtRBP1) and localizes normally nuclear localized MtRBP protein to cytoplasm [70] and regulates translation.

Computational and experimental approaches, like ribosome profiling and mass spectrometry, identified many small peptides, also known as microproteins/micropeptides of length < 100 amino acids, and coded by non-coding RNA. Because genome annotations were based on the criteria that the protein should have >100 amino acids, the ORF should be longer than 300 nucleotides, and should be flanked by 5′ and 3′ untranslated regions with AUG as the initiation codon; the small open reading frames (sORFs), which code for microproteins/micropeptides less than 100 amino acids, were not regarded as true ORFs. The proteolytic cleavage of longer polypeptides does not produce these microproteins/micropeptides. Rather, these are peptides that are translated from short ORFs found in sequences that are typically referred to as non-coding. Short ORFs (sORFs) typically consist of a sequence segment that starts with a start codon and ends with a stop codon; they differ from ORFs coding for proteins only in size. The experimental methods used to confirm the computationally predicted micropeptide coded by a lncRNA include (i) ribosome profiling, (ii) the mass spectrometric determination of the peptides, (iii) the in vitro translation of sORF, (iv) the production of specific antibodies and the detection of the peptide by a Western blot analysis, (v) cloning the sORF in a tagged vector, expressed in vivo, and the detection of the peptide; and (vi) the deletion/overexpression of the micropeptide in models to identify the functional changes [64,65]. Some lncRNAs might, thus, have bi-functional activity; lncRNAs might interact directly with their target and execute their functions, or the same lncRNA might code for a microprotein, and the microprotein, by interacting with its target perform its functions [66] (Figure 3). The targets of micropeptides and the host lncRNAs that encode them are not necessarily the same. LncRNAs encoding the micropeptides may interact with targets like proteins, nucleic acids (DNA and other RNAs), and even lipids, and regulate their functions. In humans, the binding of micropeptides with proteins or RNA may regulate protein phosphorylation, transcription, translation, protein degradation, and membrane regulation. LncRNA directly interacts with chromatin, proteins, DNA, microRNA, and mRNA; regulates transcription and splicing, and sequesters microRNA to increase the targets of miRNA, mRNA stability, translation, etc. For example, LINC00665, which codes for a micropeptide CIP2A-BP of 52-amino acid peptide, targets protein phosphorylation, while lincRNA interacts with miR-379-5p to enhance the levels of its target LIN2B [67].

There are several micropeptides experimentally identified with known functions. The first sORF encoded peptide of 10 amino acids long was identified from the lncRNA ENOD40 in soybean [71]. Subsequently, two micropeptides with lengths of 12 and 24 amino acids are encoded by the legume and non-legume lncRNA ENOD40, which have been identified. The auxin response and nodule growth in the Leguminous species were regulated by the ENOD40-coded micropeptides [69]. The micropeptide ROTUNDIFOLIA (53 amino acids) is encoded by the ROT4 ORF in Arabidopsis. ROT4’s function in leaf and flower formation is demonstrated by the small leaves and floral organs that arise from overexpression [72]. A small peptide (36 amino acids) coded by POLARIS (PLS), a lncRNA of about 500 nucleotides long, was identified in Arabidopsis. Normal vascular development, root growth, and auxin and cytokinin responses all depend on the micropeptide that PLS encodes [73,74]. The *Arabidopsis thaliana* pri-miR165a and alfalfa (*Medicago truncatula*) pri-miR171b encode the short peptides ath-miPEP165a and mtr-miPEP171b, respectively. When plants are exposed to synthetic miPEP171b and miPEP165a peptides, mature miR171b and miR165a accumulate, which inhibits the formation of lateral roots and promotes the growth of main roots. This observation suggests that the actions of these small peptides are through enhancing transcription. These small peptides might have agronomical applications [75]. A similar result has also been obtained with pri-miR858a in *A. thaliana*. The expression of mature miR858a and its target genes is demonstrated to be regulated by the primary pri-miR858a, which encodes the short peptide miPEP858a. Additionally, it was demonstrated that plants with miPEP858a overexpression and editing changed the formation and levels of flavonoids by altering the expression of genes linked to auxin signaling and the phenylpropanoid pathway [76]. Thousands of sORFs in lncRNAs have the ability to translate into microproteins/micropeptides or peptides. The moss *Physcomitrium patens* has lncRNAs that encode peptides. PSEP3 is one of these lncRNAs; it codes for a 57-amino acid peptide containing proline-enriched low-sequence-complexity regions (LCRs). The protonemata and gametophores of *P. patens* translate PSEP3, and protonemata growth is impacted by its overexpression (OE line) or deletion (KO line). The LC-MS/MS analysis of *G. max* and *G. sojae* root tissues identified 153 micropeptides encoded by 179 lncRNAs. The function of the identified micropeptides was predicted based on the co-expression of protein genes with the micropeptides. These micropeptides have been identified to co-express with the protein-coding genes linked to the production of metabolite and energy precursors, photosynthesis, light reaction, ATP synthesis coupled with electron transport, and defense gene control. This finding suggests the potential functions of the discovered micropeptides in the aforementioned processes [77]. A comprehensive analysis of lncRNA-coded sORFs from the moss *P. patens* has been reported. Approximately 5000 of the 70,000 transcribed sORFs in the moss *P. patens* were preserved across several species. Since most sORFs seem to be evolutionary young, they may be a significant source of functional innovation. The transcriptional level of conserved sORFs is generally higher than that of non-conserved sORFs. A proteome analysis confirmed the translation of 82 new species-specific sORFs. Many conserved sORFs with transmembrane domains or low-complexity regions (LCRs) have been identified, and the biological roles of a few of these LCR-sORFs were experimentally demonstrated. Therefore, a significant functionally heterogeneous part of the plant proteome are microproteins encoded by sORFs [78]. The mutant lines with PSEP3 overexpression (OE) and knockout (KO) were used for the quantitative proteomic analysis. For the iTRAQ-based proteome studies, seven-day-old protonemata from both the wild type and mutant lines (KO and OE) were used. It was revealed that the knockout of PSEP3 modulates the expression of 2873 proteins; 167 differentially expressed proteins were identified after he induction of PSEP3 overexpression. In PSEP3 KO plants, several photosynthetic proteins were downregulated, but, in PSEP3 OE plants, they were upregulated. While dynamin-related protein 1C was increased in PSEP3 OE plants, differently expressed proteins such as catalase, xyloglucan endo-transglycosylase, and metacaspase-4-related protein were downregulated in PSEP3 KO plants. These proteins play a role in organelle function, cell death, cell wall structure, and antioxidant defense [79]. Numerous putative sORFs from 2743 lncRNAs have been identified using Ribo-Seq data from 226 *A. thaliana* samples and catalogued in the AthRiboNC database (https://bis.zju.edu.cn/athribonc/, accessed on 2 March 2025) [80]. Non-coding transcript RPG has recently been shown to code for a small peptide microRPG1 of 31 amino acids in maize. It is also shown that microRPG1 regulates the expression of the Ethylene-insensitive 3-like 1 and 3 genes and modulates Kernel dehydration [81].

Micropeptides coding from lncRNA are observed in diverse species, including plants and humans, and studies on the role of micropeptides are an evolving area of active research. The results revealed so far from the diverse studies in animals and plants revealed a substantial number of lncRNAs code for micropeptides conserved well within the genus in soya beans *Drosophila melanogaster* even with other plant species [77,78,82], which indicates that micropeptides coding from lncRNA are not rare but a general phenomenon with which to diversify the genomic functions. The diversification of function of lncRNAs through coding small peptides is also evident from the dual functions of lncRNA [66]. In summary, the generation of precursors of metabolites and energy, photosynthesis, light reaction, ATP-synthesis-coupled electron transport, defense, antioxidant defense, cell wall structure, cell death, organelle functioning, kernel dehydration, and gene expression are all demonstrated experimentally to be regulated by small peptides encoded by the lncRNAs or primary transcripts of microRNAs from a few plants. A large-scale mass spectrometric analysis, ribosome profiling, and computational analysis identified or predicted hundreds, if not thousands, of microproteins coded by non-coding RNAs in plants. Several current reviews are available to describe the details of sORFs/micropeptides coded by non-coding RNAs, especially the lncRNA in different plants and their possible roles in biological functions [64,83,84]. The majority of sORFs/micropeptides identified and characterized play important roles in plant development and the stress response [64], but only in few cases have their functions been confirmed by experiments so far.

### 2.6. Subcellular Localization of Plant lncRNAs

The localization of plant lncRNA is a critical factor for their functions. Subcellular localization can enable distinct functions of the lncRNA by interactions with different interacting partners and targets of action. Animal lncRNAs are localized in the nucleus, cytoplasm, ribosomes, mitochondria, and extracellular microvesicles (http://www.rnalocate.org/ or http://www.rna-society.org/rnalocate/, accessed on 2 March 2025 [85]). Plant lncRNAs are primarily observed in the nucleus and cytoplasm and perform diverse functions in both compartments, including gene regulation, stress responses, and development. Some lncRNAs might be present both in the cytoplasm and nucleus; depending on their locations, their functions could be different. Nuclear lncRNAs differ significantly among *A. thaliana*, *O. sativa*, and *Z. mays*, according to estimates of the proportions of lncRNAs in the nucleus, cytoplasm, ribosomes, and exosomes (Table 4) [86]. In *A. thaliana*, *O. sativa*, and *Z. mays*, the cytoplasmic/nuclear (C/N) ratio of total lncRNAs was 0.48, 0.71, and 0.97, respectively (Figure 2B of the published paper [86]). While lncRNAs are equally distributed in the cytoplasm and nucleus of Z. mays, they are nearly twice as localized in the nucleus of *A. thaliana*. This suggests that either the abundance and subcellular localization of lncRNAs may differ among plant species, or their distribution in the cytoplasm and nucleus may be dynamic. According to initial studies using human cell lines, just 4% of lncRNAs were localized in the cytoplasm, whereas roughly 17% of them did so in the nucleus [87]. However, according to the statistics in RNALocate v3.0 [85], approximately 32%, 65%, and 3% of lncRNAs are found in the ribosome, nucleus, and cytoplasm, respectively. Further research is required to ascertain if the proportion of nuclear and cytoplasmic RNAs varies with the growth conditions or in species-dependent fashions, given the enormous number of lncRNAs in diverse plant species. The nuclear localization of lncRNAs suggests that they can splice and associate with DNA or chromatin to carry out nuclear processes, including transcription control. By changing the stability or location of the interacting partners, lncRNA interactions with mRNAs, proteins, or microRNAs in the cytoplasm may change how protein-coding genes function.

### 2.7. Tissue-Specific Expression of lncRNAs

In contrast to the mRNAs of protein-coding genes, lncRNAs are often expressed in a tissue-specific way. Out of 3718 lncRNAs, 1208 lncRNAs—also referred to as lincRNAs—that are transcribed from the intergenic regions of Arabidopsis exhibit a variable expression in the flowers, leaves, and roots. A total of 212 lncRNAs were found to be expressed preferentially in flowers, while 362 and 272 lncRNAs were found to be expressed preferentially in leaves and roots, respectively. For example, At4NC047210 was expressed highly in flowers, while, in leaves and roots, the expressions were much lower. At2NC044550 was highly expressed in leaves compared to in flowers and roots. At1NC018710 was expressed highly in roots compared to in flowers and leaves. Altogether, 212 lncRNAs are preferentially expressed in flowers, 362 in leaves, and 272 in roots [52].

**Figure 4:**
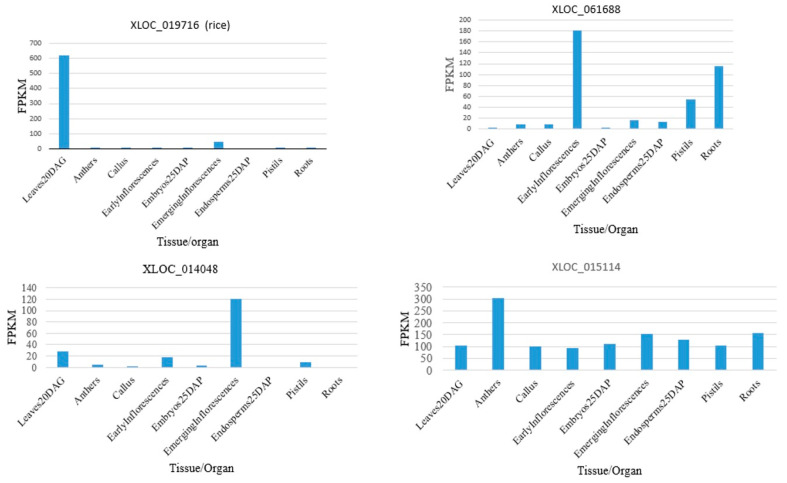
Expression of representative lncRNAs in different tissues. The result was taken from the additional file 2 of the published paper [88]. The acronym for “fragments per kilobase of transcript per million mapped reads” (FPKMs) is used to normalize RNA-seq data and quantify gene expression.

The expression patterns of rice lncRNAs vary by tissue and stage. Rice anthers, pistils, and seeds harvested five days after pollination, and shoots harvested fourteen days after germination, were found to have a differential expression of lncRNAs, including intergenic and natural antisense RNAs. It was observed that more than 2000 lncRNAs were expressed differentially in different tissues and other conditions (additional file 2 of the published paper [88]). Among the validated lncRNAs, XLOC_018316, XLOC_057981, and XLOC_040350 were expressed almost exclusively in anthers, XLOC_037529 was expressed only in embryos, XLOC_016182 was expressed in seeds for 5 days, and XLOC_045319 was expressed highly in the embryos and callus. From the additional file 2 of the published paper [88], it was shown that the lncRNA XLOC_019716 was expressed in leaves 20 days after germination, XLOC_061688 in early inflorescences, XLOC_014048 in emerging inflorescences, and XLOC_042841 was expressed maximally among the tissues studied. Some lncRNAs in rice were expressed almost equally in different tissues. For example, XLOC_015114 was expressed maximally in anthers, although the expression of the lncRNA was high in other tissues (Figure 4).

About 54% of the 20,163 putative lncRNAs—of which 1704 are regarded as high-confidence in maize—were found in just one tissue (with at least four RNA-seq reads detected). Five or more tissues contained about 10% of the lncRNAs. More instances of tissue-specific expression were found in the female reproductive tissues, such as embryo sacs, and the male reproductive tissues, such as immature tassel, anther, and pollen [89]. In cotton (*Gossypium* spp.), 50,566 lncRNA transcripts coded from the intergenic 30,550 genomic loci and 5826 transcripts from the 4718 natural antisense (NAT) loci are identified. Using this data, tissue specificity scores, like the Jensen–Shannon divergence (JS score), which quantifies how much a gene’s expression is concentrated in a specific tissue compared to others and ranging from 0 (ubiquitous expression) to 1 (tissue-specific), the tissue-specific expressions in the root, hypocotyl, leaf, flowers, etc. are determined. Compared to the percentage of protein-coding transcripts (18%) that were tissue-preferentially expressed throughout the tissues, 42% of lncRNAs were coded from the intergenic loci, and 51% of NAT lncRNAs were found to be tissue-preferentially expressed, using a JS score of 0.5 as a cut-off. The largest number of tissue-preferential genes, including 3140 protein-coding genes, 3925 intergenic-region-coded lncRNAs, and 787 natural antisense transcripts, were expressed in the anther, according to an additional quantitative study. In contrast, only a small number of particular genes were expressed by cotton fibers 20 days after anthesis (973 protein-coding transcripts, 852 intergenic-coded lncRNAs, and 230 NAT lncRNAs). This finding suggests that cotton lncRNAs are also expressed preferentially in specific tissues [90]. In the updated version of the database CANTATAdb (version 3.0), 112,980 lncRNAs are reported to be expressed in a particular plant tissue or embryos specifically. A summary of the number of lncRNAs explicitly expressed in the stem, seed, leaf, root, fruit, flower, and embryo in 80 different plants is depicted in Supplementary Table S2 of the published paper [59]. For example, in *Papaver somniferum* (*Opium poppy*), 6389 lncRNAs are specifically expressed in th leaves, and 4298 lncRNAs are expressed in the roots [59]. The tissue-specific expression of the lncRNA indicates that lncRNAs might be involved in functions related to the specific tissue/organ.

### 2.8. Epigenetic Regulation of lncRNA in Plants

**Figure 5:**
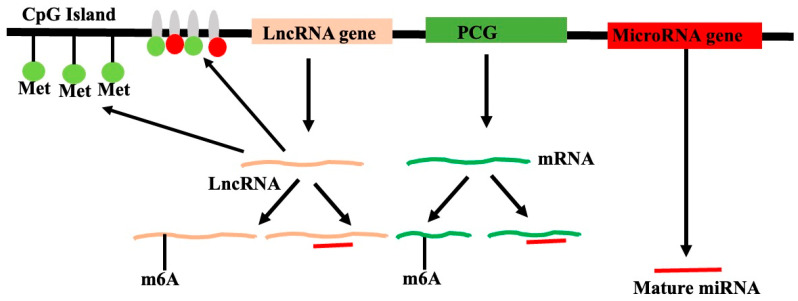
Epigenetic regulation of protein-coding genes, lncRNA gene, and microRNA genes (shown by different colors) by the core histones of the nucleosome (grey color, vertical filled elliptical shapes) modification like methylation (green filled circle), acetylation (red filled circle), and methylation of CpG islands (vertical lines with filled green circle). LncRNA can interact with DNA and chromatin to induce epigenetic changes, resulting in altered transcription. N6-adenine methylation (m6A) of transcripts might alter the stability of transcripts. Similarly, mature microRNA, by interacting with the transcripts, might alter the levels of transcripts. Both m6A and microRNA modify the expression of genes at the post-transcriptional level.

Epigenetic regulation is the control of the gene expression without altering the DNA sequences. Such regulation is carried out by (a) DNA methylation, (b) the modification of histones involved in chromatin formation by methylation, acetylation, or other modifications of core histones that alter the chromatin states, (c) the N6-adenine methylation (m6A) of the transcripts, and (d) microRNA at the post-transcription stage. The DNA methylation and chromatin state regulate the expression during transcription, while N6-adenine methylation and microRNA regulate the expression at post-transcription levels. The regulation of the gene expression by lncRNA through its interactions with the DNA or chromatin/nucleosome could also be referred to as epigenetic regulation (Figure 5).

The CpG, CpHpG, and CpHpH sites—where H might be A, C, or T—are where the methylation of DNA occurs most frequently in plants. This type of alteration is observed in excess at the transposable elements (TEs), repeat sequences, and heterochromatin regions, and is crucial in transcriptional gene silencing (TGS), which is the process of reducing the expression of these genes. When DNA methylation is primarily found in the promoters or enhancers of genes, it causes the genes to be repressed. Alternative splicing and alternative polyadenylation are two mRNA processes that might be impacted by the methylation of TEs in introns and repeats [91]. DNA methylation, primarily in the CpG context, may also exist in the gene bodies of many plant species; the impact of this change on the gene expression is unclear [92]. A review of the enzymes and proteins involved in DNA methylation, DNA methylation maintenance, and DNA demethylation in plants has been published [93].

#### 2.8.1. Epigenetic Regulation of lncRNA Expression

Like protein-coding genes, lncRNAs are under epigenetic regulation in plants and animals [93]. Many studies have shown that DNA methylation levels are negatively correlated with the expression of protein-coding genes. The connection between DNA methylation and lncRNA expression has not received much attention in research. The expression of lncRNAs increased significantly in plants (*Arabidopsis*, rice, tomato, and maize) with DNA methylation defects due to mutations in the genes involved in DNA methylation. This finding shows DNA methylation controls the expression of lncRNAs [94]. RNA-seq and bisulfite-converted DNA sequencing from cotton petals were performed to examine the ways in which DNA methylation changes the gene expression. The expression of both lncRNA and protein-coding genes correlated with DNA methylation levels. The DNA methylation levels were low for genes with high expression levels and low for genes with high levels of methylation. This finding suggests a negative relationship between DNA methylation and gene expression for both lncRNAs and protein-coding genes. Compared to lncRNAs, protein-coding genes displayed a more tightly distributed pattern of methylation levels in each of the CG, CHG, and CHH contexts for gene body methylation. While there was no appreciable correlation between upstream methylation and gene expression, the gene body methylation of lncRNAs in each methylation environment differed significantly from that of protein-coding genes. These findings imply that, in general, gene body methylation affects the protein-coding gene expression more strongly than lncRNAs [90]. *Populus simonii* (poplar) lncRNAs TCONS_00268512, TCONS_00020674, TCONS_00124808, and TCONS_00201294 have been found to exhibit promoter methylation, and TCONS_00177789’s gene body methylation has been found to dynamically modulate the expression in response to abiotic stress [95]. It has been observed that gene body methylation in intragenic antisense transcripts inhibits gene expression; in Arabidopsis, aberrant intragenic transcripts are repressed by both H1 and DNA methylation [96]. High-salinity environments frequently cause DNA methylation, which is linked to modifications in the expression of genes that code for proteins and non-coding RNA, such as lncRNA, which increases the plant resistance to salt. Numerous lncRNAs that are differently expressed have been found in the roots and leaves of tomatoes, salt-tolerant rice, and tobacco. Similar to genes that code for proteins, these lncRNAs are probably controlled by DNA methylation [97].

#### 2.8.2. LncRNA as Epigenetic Regulators

Plant lncRNAs have the ability to alter DNA methylation, the most prevalent type of epigenetic control. By altering the CpG methylation status of DNA, LncRNA can control gene expression; the methylation of the CpG in the gene regulatory regions changes the expression of the gene. The formation of the “R loop” due to the lncRNA–DNA interaction has been shown to protect the promoters from CpG methylation by DNA methylase and facilitate the transcription of the targets of the lncRNA [98]. Due in part to their capacity to regulate histone changes that impact the chromatin structure and gene accessibility, lncRNAs are crucial in controlling the gene expression in plants. To control the transcription of the downstream target genes, lncRNAs interact with the proteins in the transcription complex, such as the mediators, transcription factors, and transcription repressors. For instance, in Arabidopsis, lncRNA ELENA1 increases the expression of genes involved in the innate immune response through its interaction with Mediator subunit 19a (MED19a). LncRNA HID1 functions as a transcriptional repressor by interacting with chromatin in the region included in the first intron of the 5ʹ UTR of its target gene PIF3. Several other lncRNAs might affect the target genes by acting as chromatin modifiers [99]. Proteins belonging to the Polycomb Group (PcG) are essential modulators of gene expression and play a role in many processes, including development. PcG assembles into complexes that alter the target genes’ post-translational histone tails. In plants, the Polycomb Repressive Complex 2 (PRC2) complex includes the histone H3K27 trimethyl transferase CLF as a catalytic member. The PRC1-like components LHP1 and AtRING1 are then recruited with the assistance of modified histone H3K27me3. In addition, the Trithorax H3K4 methyltransferase ARABIDOPSIS TRITHORAX-LIKE PROTEIN 1 (ATX1) mediates the establishment of H3K4me3. It is interesting to note that different lncRNAs have been linked to the post-translational changes of the histones at the target loci, which are facilitated by the addition or subtraction of PcG, and Trithorax proteins. LncRNAs may also post-transcriptionally control gene expressions through their interactions with microRNAs and splicing factors. As we will see in the following section, lncRNAs also play a role in RNA-dependent DNA methylation, which epigenetically controls gene expression [100].

#### 2.8.3. RNA-Dependent DNA Methylation (RdDM)

**Figure 6:**
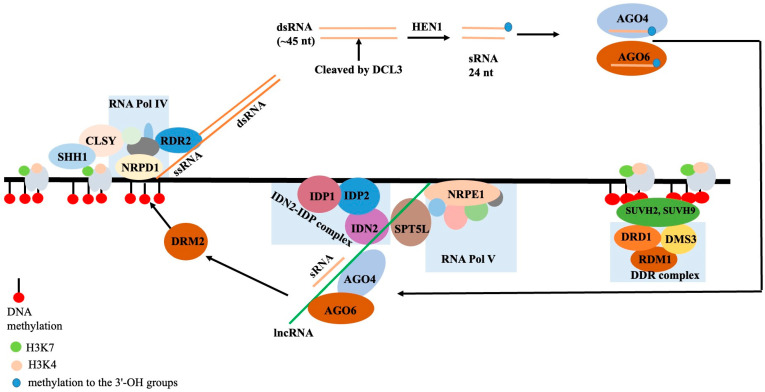
Key mechanism of actions in the plant’s canonical RdDM pathway. See the text for further information.

The mechanism by which non-coding RNA molecules directly methylate DNA to particular sequences is referred to as RNA-directed DNA methylation (RdDM) in plants. RdDM, the primary mechanism catalyzing de novo methylation in plants, has been better understood mechanistically as a result of in vitro research and reconstituted enzyme systems [101]. Plant-specific Pol V-transcribed lncRNAs take part in RdDM, a process in which lncRNAs direct DNA methylation to specific locations, frequently suppressing transposable elements and repetitive sequences. Small transposable elements (TEs) and TE fragments close to genes are the primary targets of RdDM. These genes are permissive of gene expression and are typically found in open, accessible euchromatic sections of the genome. In these areas, the “active” chromatin state has a tendency to transfer from expressed genes to adjacent repressed regions, such as TEs, and can trigger the activation and transposition of these TEs. By keeping TEs in these ordinarily euchromatic areas in a silent, repressive heterochromatic state, RdDM prevents the propagation of active chromatin. RdDM activity then attracts other pathways that contribute to the establishment and spread of the heterochromatic, quiet state. Angiosperms, or flowering plants, have a well-characterized RdDM pathway, particularly in *A. thaliana.* Other plant families, including gymnosperms and ferns, have been found to have conserved elements of the RdDM pathway linked to short RNAs (sRNAs). The canonical RdDM acts to enhance the pre-existing DNA methylation patterns at heterochromatic regions that are already DNA-methylated by preferentially recruiting to these loci. The CpG, CpHpG, and CpHpH sites can all be methylated by RdDM, where H can be any nucleotide other than G. The two primary steps of the RdDM pathway are (a) sRNA synthesis and (b) the sRNA-assisted recruitment of DNA methylation machinery to particular target loci in the DNA. Plant-specific RNA Polymerase IV complex (NRPD1 is the largest component of the complex) is recruited to the silent heterochromatin through its interaction with CLSY proteins and SAWADEE homeodomain homolog 1 (SHH1) to initiate the transcription of short single-stranded RNAs (~30 to 45 nucleotides in length), the precursor for a single sRNA. RNA-directed RNA polymerase 2 (RDR2) interacts with Pol IV and co-transcribes single-stranded RNA into double-stranded RNA. In the first step, NRPD1 is the largest subunit of the Pol IV complex. The endoribonuclease Dicer-like 3 (DCL3) splits double-stranded RNA into 24 nucleotide (nt) sRNAs. By methylating the 3′-OH groups, the RNA methylase HEN1 may stabilize sRNAs. Each 24 nt double-stranded sRNA strand is loaded into one of the Argonaute proteins, primarily AGO4 and AGO6. The AGO-sRNA duplex binds the complementary RNA sequences that RNA Polymerase V transcribes. RNA Polymerase V has found and coded thousands of lncRNAs [102]. By interacting with the Pol V subunit NRPE, the Involved in de novo 2-IDN2 Paralog (IDN2-IDP) complex, and the suppressor of Ty insertion 5-like (SPT5L), these lncRNAs function as “scaffolds”. As a result, Domains Rearranged Methyltransferase 2 (DRM2), an enzyme that methylates neighboring DNA, is recruited to the complex. Transgene silencing, the abiotic and biotic stress response, development and reproduction, genome stability, short- and long-range signaling, and other processes have all been linked to EdDM [103]. Simplified steps in the canonical RdDM pathway are shown in Figure 6.

#### 2.8.4. N6-Methyladenosine (m6A) Modification of RNA: Post-Transcriptional Epigenetic Modifications

As a dynamic and reversible epigenetic mark, N6-methyladenosine (m6A) is a common post-transcriptional RNA modification in plants that is essential for controlling gene expression, plant growth, and responses to biotic and abiotic stressors. m6A is the most prevalent chemical modification found in eukaryotic mRNAs, such as those found in plants, among the various RNA chemical modifications that have been detected, including mRNA, rRNA, tRNA, microRNA (miRNA), and lncRNA. In a plant cell, between 50 and 60 percent of the transcripts are changed at m6A; the most frequent modification is one, followed by two or four. In both plants and mammals, the m6A markers are found in 3Ϲ-UTRs and near stop codons. Methyltransferases and a few auxiliary proteins, together called “writers”, are responsible for adding the m6A mark. MTA, MTB, VIRILIZER, FIP37, HAKAI, HAKAI-interacting zinc-finger protein 2, and FIONA1 are among the writers identified in various plants. Demethylases, sometimes referred to as “erasers”, eliminate the m6A mark, whereas RNA-binding proteins, sometimes called “readers”, interpret it. In various plants, erasers include the proteins ALKBH2, ALKBH8B, LKBH9, ALKBH9B, ALKBH9C, and ALKBH10B. RNA-binding proteins ECT1, ECT2/3/4, ECT8, ECT9, ECT12, YTP2, CPSF30-L, FLK, YTP8/9, and YTH07 are among the readers [104,105].

**Figure 7:**
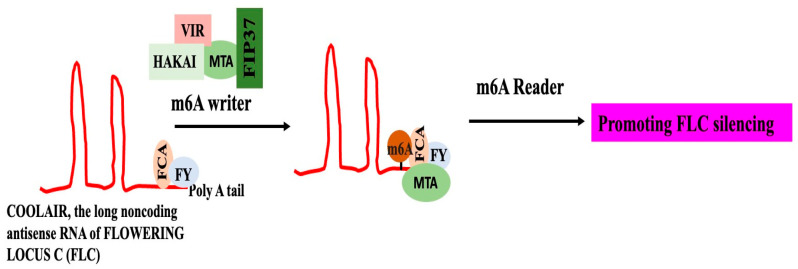
Flowering locus C silencing in Arabidopsis, by m6A modification of COOLAIR, the long non-coding antisense RNA of FLOWERING LOCUS C (FLC). LncRNA COOLAIR can be methylated by the m6A writer MTA and accessory subunits such as FKBP12-interacting protein 37 kDa (FIP37), VIRILIZER (VIR), and an E3 ubiquitin-protein ligase HAKAI. This results in m6A marks on COOLAIR, which alters its conformation and improves its interaction with FLOWERING CONTROL LOCUS A (FCA) and the 3′-RNA processing factor FY. FLC repression and early blooming result from chromatin silencing caused by an unidentified m6A reader [104].

Animals, including humans, have been shown to exhibit the function of m6A alteration in controlling the expression of lncRNAs [106]. Only a small number of plants have been examined for the function of m6A in lncRNA regulation. The lncRNA COOLAIR’s m6A alteration is crucial for controlling flowering timing and gene regulation in Arabidopsis. When COOLAIR undergoes this m6A alteration, it interacts more effectively with FLOWERING CONTROL LOCUS A and the 3′-RNA processing factor FY, which suppresses the production of FLOWERING LOCUS C (FLC) (Figure 7). It is suggested that the m6A alteration will cause conformational changes in COOLAIR, increasing its attraction to proteins that interact with it [107].

Arabidopsis wild-type (Columbia-0), 2381 m6A modification sites on lncRNAs have been identified. The levels of m6A were lower in lncRNAs than in mRNAs. The m6A modification boosted the quantity of lncRNAs. A comparison of the abundance of lncRNAs in 2-week seedlings (vegetative stage) and 5-week floral buds (reproductive stage) revealed that 289 annotated lncRNAs and 513 novel lncRNAs were found in the reproductive stage, whereas 226 annotated lncRNAs and 451 novel lncRNAs were found in the vegetative stage samples. There were 484 lncRNAs that were expressed in both the vegetative and reproductive stages, 193 that were expressed primarily in the vegetative stage, and 318 that were expressed specifically in the reproductive stage. In both 2-week seedling and 5-week floral bud samples, lncRNAs showed a total of 353 unique m6A-modified sites, which accounted for 26% of all methylation sites. Additionally, lncRNAs were found to have 676 methylation sites specific to floral buds and 324 methylated sites specific to seedlings. This finding demonstrates how m6A alterations of lncRNAs exhibit dynamic expression patterns as they go from the vegetative to the reproductive stages. Furthermore, favorable relationship between the methylation variations and variations in lncRNA expression across the developmental phases was revealed. During the growth of the Arabidopsis stage, epigenetic modification and post-transcriptional processing may influence the expression of lncRNAs and their roles [108].

## 3. Functions of Plant lncRNAs

**Figure 8:**
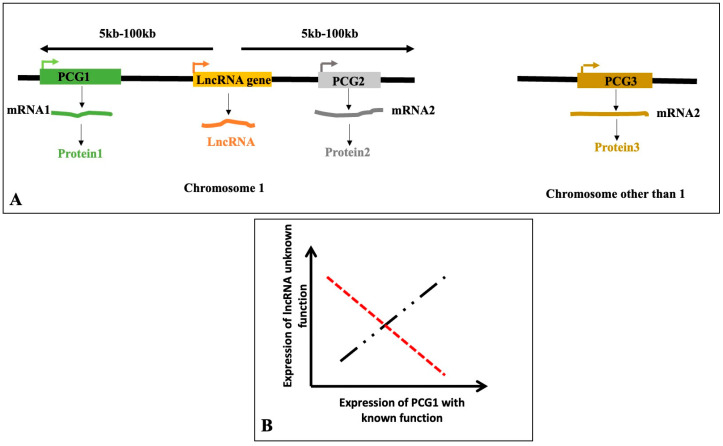
Principles for the prediction of functions of lncRNAs in general and stress-induced lncRNAs in particular from the known functions of nearby protein-coding genes (**A**); the lncRNA might interact with protein-coding gene 1 (PCG1) or PCG2, also known as cis- interaction. The lncRNA might also interact with PCG3 residing on different chromosomes than chromosome 1 (trans-interaction). The lncRNA might also co-express with protein-coding genes (**B**). From the known function(s) of the protein-coding genes (PCG1, PCG2, or PCG3), the function(s) of the lncRNA are inferred. The black dotted line represents a hypothetical positive correlation while red dotted line represents a hypothetical negative correlation between the expression of lncRNA and PCG.

Hundreds of thousands of lncRNA sequences have been found in a variety of plant species, because of high-throughput RNA sequencing and potent bioinformatics tools. However, only a handful of lncRNAs have been shown to possess definitive physiological functions. Plant lncRNAs are involved in a broad spectrum of biological functions such as development (plant height development, e.g., GARR2; root development, e.g., ASCO; and flowering regulation, e.g., COOLAIR and COLDAIR), nutrient signaling (e.g., IPS1), and stress responses (e.g., SVALKA and PILNCR1), and acting through diverse mechanisms including chromatin remodeling, miRNA sequestration, and transcriptional repression [Supplementary Table S1]. There are different approaches with which to determine the functions of lncRNAs. The functions of the lncRNA are generally inferred from the functions of the protein-coding genes they physically interact with or the lncRNA “co-expressed” with the protein-coding genes. LncRNAs and protein-coding genes are considered to be co-expressed and likely to participate in the same physiological process when their expression patterns show a statistically significant positive or negative correlation. Since closely located genes on chromosomes are frequently co-expressed, it is likely that the same regulators control the co-expressed genes. Since lncRNAs are known to interact with and affect surrounding protein-coding genes, in certain situations, the functions of the lncRNAs are inferred by using closely spaced protein-coding genes (upstream and downstream positions within 5–100 kb, as utilized by various authors) [90] (Figure 8).

By recruiting or displacing transcription factors at the promoters of nearby genes, cis-acting lncRNAs often control the transcription of genes located in close chromosomal proximity [29]. The distance between lncRNA and protein-coding genes used by different investigators varies. Protein-coding genes, approximately 100 kb upstream or downstream of the genomic position of the lncRNA of interest, have been used by many investigators to infer the functions of the lncRNAs [109]. High-throughput and low-throughput assays are available to determine the lncRNA–protein interactions. In the low-throughput hypothesis-based assay, crosslinking between RNA and the protein of interest is immunoprecipitated with the antibody against the protein after cross-linking. The specific lncRNA is detected in the immunoprecipitated solution by reverse-transcription-mediated PCR. On the other hand, immunoprecipitated RNA can be sequenced to determine which lncRNAs interact with the specific protein. Directly determining the biological function of lncRNAs can be achieved from the transgenic plants over-expressing the lncRNA or in plants where the lncRNA is knocked out/or mutated, and the phenotypes of the transgenic plants were altered.

### Physiological Functions of lncRNAs in Plants

**Figure 9:**
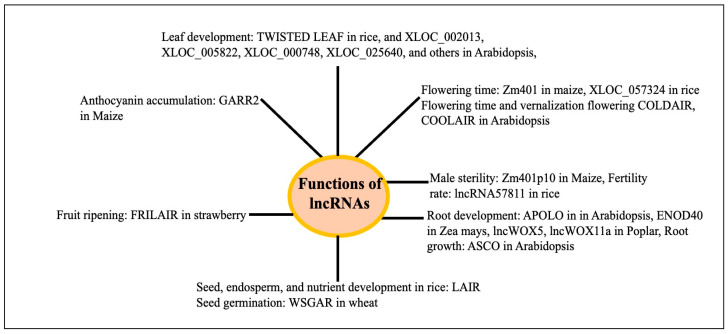
Growth and developmental functions of lncRNAs in different plants. See the main text and Supplementary Table S1 for details and references.

Based on the principles described above, diverse physiological functions of lncRNAs have been inferred or identified. Plant lncRNAs are involved in growth and development across different organs like the root, leaf, seed/endosperm, seed germination, and environmental stresses like drought, salt, flood, heat, cold, etc., and have recently been reviewed [25,28,110,111] [Supplementary Table S1]. The LncRNA ASCO controls root development in *Arabidopsis* by participating in splicing activity at the molecular level [112]. Several lncRNAs have been associated with the development of roots (APOLO, lncWOX11a, and lncWOX5), leaves (TWISTED LEAF, XLOC_002013, XLOC_005822, XLOC_000748, XLOC_025640, and others 442 developmental age-related lncRNAs), and seeds, endosperm, and nutrients (LAIR) in different plants [28]. LncRNAs (746) were observed to exhibit a variable expression in *Arabidopsis* at various phases of leaf development. Of these lncRNAs, 28 were engaged in leaf development and were a member of the leaf development regulatory network mediated by interactions with either circular RNAs or microRNAs [113]. The PSY1 gene is trans-spliced by the lncRNA ACoS-AS1. The splicing of PSY1 causes yellow fruits in tomatoes [114]. The maturation of strawberries is influenced by fruit-ripening-related long intergenic RNA (FRILAIR) [115]. The lncRNA WSGAR in wheat interacts with miR9678 and regulates wheat seed germination by regulating the Gibberellin signaling pathway [116]. In *Arabidopsis*, the lncRNA ASCO binds to SmD1b and PRP8a, two components of the spliceosome. The overaccumulation of ASCO inhibits PRP8a’s ability to recognize particular transcripts linked to flagellin. When ASCO is knocked down in Arabidopsis, many genes exhibit differential splicing, suggesting that ASCO may play a role in splicing [117]. Decreased expressions of lncRNAs during seed aging were associated with flavonoid biosynthesis, energy metabolism like starch and sucrose metabolism, nitrogen metabolism, secondary metabolism, and others in rice [118]. Abnormal leaves are produced in rice when lncRNA TWISTEDLEAF (TL), which is transcribed from the opposite strand of the R2R3 MYB transcription factor gene locus (OsMYB60), is silenced. In rice, the overexpression of lncRNA57811 reduces the seed-setting rate and fertility [119]. The database EVLncRNAs 3.0 (https://www.sdklab-biophysics-dzu.net/EVLncRNAs3/, accessed on 2 March 2025 [120]) describes the biological processes and molecular functions associated with lncRNAs of different species, including many plants, mainly from the published low-throughput experiments. The biological processes and molecular functions of about 516 lncRNA from 66 plant species are catalogued. LncRNA-associated biological processes include vernalization flowering, flowering time, fruit ripening, leaf shape, branch growth, root development, seed and pod development, grain yield (rice), and many others. A summary of the different physiological functions of the lncRNAs in different plants is shown in Figure 9. Several reviews exist on the functions of the plant lncRNA using the approaches mentioned above [110,111].

## 4. Mechanisms of Action of lncRNAs

**Figure 10:**
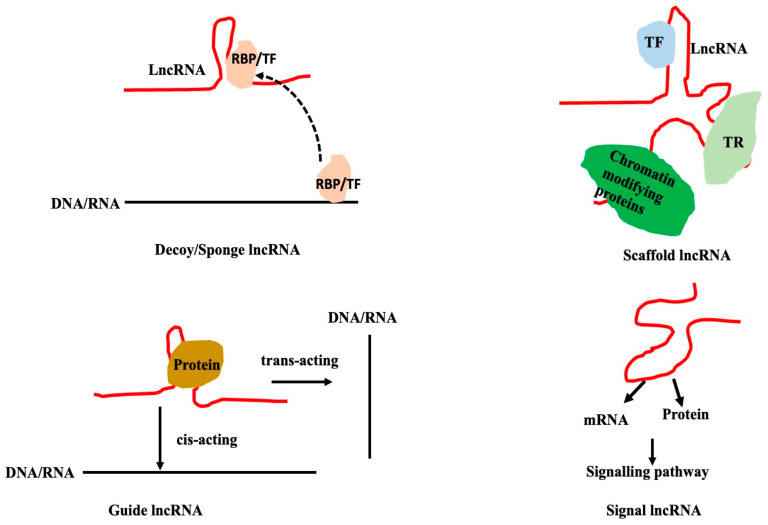
Classifications of lncRNA based on the mode of action of lncRNA. The interaction of lncRNA with RNA-binding proteins or transcription factors/repressors might prevent the binding of these proteins with their targets (shown as a broken line, top left corner). The interaction of lncRNAs with microRNAs prevents microRNAs from interacting with their mRNA targets; in both cases, lncRNAs act as decoys. LncRNA can serve as a scaffold and a platform for the recruitment of numerous proteins and protein complexes. LncRNA may serve as a guide by interacting with proteins and mRNAs to lead them to the target regions for its activity.

LncRNAs can bind to a variety of partners like DNA (chromatin or promoters, and enhancers), mRNA, microRNAs, and proteins, and can regulate a wide range of biological activities. LncRNA alters the expression of genes that code for proteins, the stability of mRNA or proteins, and the localization and trafficking of proteins. Based on the mode of action of the lncRNAs, they can loosely be classified into (a) decoy/sponge lncRNAs, (b) scaffold lncRNAs, (c) guide lncRNAs, and (d) signal lncRNAs (Figure 10). LncRNAs acting as scaffolds, guides, or decoys mostly regulate the transcription or splicing by interacting with DNA or RNA. Depending on the targets of lncRNA-interacting miRNAs, lncRNA may act as a decoy to either alter the gene expression or signalling. LncRNAs interacting with proteins/mRNAs mainly act as signal molecules. Such a classification might not be straightforward but it helps to conceptualize the processes and may not be clear.

### 4.1. Long Non-Coding RNA–MicroRNA Interaction: Decoy/Sponge lncRNAs

LncRNA binds directly to proteins like transcription factors or repressors, proteins involved in splicing, or RNA-binding proteins, and sequesters or acts as a sponge of the targets. Such binding prevents the normal functioning of the targets of lncRNA. The interaction of lncRNAs with proteins may sequester the protein. LncRNA can also bind to microRNA and prevent the microRNA from targeting their targets. There are many examples of miRNA sequestration due to interactions with lncRNAs. LncRNAs may act as decoys/sponges by interacting with proteins or microRNAs. For each of them, we provide some examples in the section that follows.

#### 4.1.1. LncRNA–Protein Interaction: lncRNAs as Decoy

**Figure 11:**
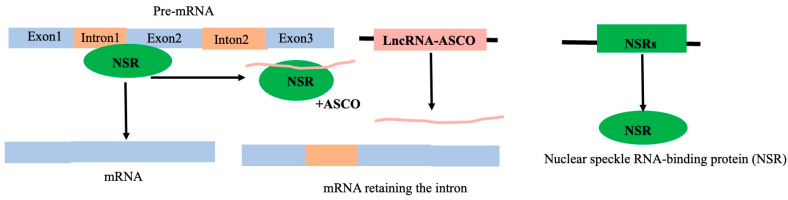
Example of a lncRNA (ASCO) acting as a decoy. The protein NSR (shown in green), interacting with other proteins necessary for splicing, binds to the intron–exon boundary pre-mRNA and is involved in the splicing process. The lncRNA ASCO can interact with NSR; such interaction prevents NSR from binding with the intron–exon boundary, resulting in a faulty transcript product with the retention of the intron [112].

Decoy lncRNAs interacting with proteins preclude the access of regulatory proteins to their targets; the targets of the regulatory proteins could be DNA or RNA. For instance, the splicing-related protein Nuclear Speckle RNA-binding protein (NSR) can interact with the lncRNA ASCO in *Arabidopsis*. The intron of the NSR target gene is retained as a result of ASCO’s interaction with NSR, which stops NSR from interacting with RNA (Figure 11) [112,121]. The lncRNA ENOD40 in *M. truncatula* can interact with MtRBP1, a homolog of nuclear speckle RNA binding protein, located in the nuclear speckle. Such an interaction with ENOD40 re-localizes MtRBP1 to the cytoplasm from the nucleus speckle, resulting in the modification of root nodule organogenesis [70,121,122]. LHP1, a part of the polycomb repressive complex 1 (PRC1), escapes from attaching to promoters and suppressing the target genes when it interacts with the lncRNA APOLO [123].

#### 4.1.2. Long Non-Coding RNA–MicroRNA Interaction

**Figure 12:**
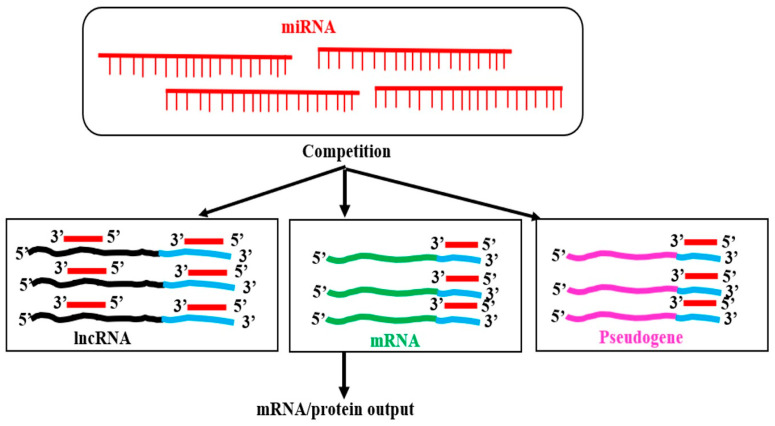
Competing endogenous RNA (ceRNA) hypothesis. By sharing microRNA response elements (MREs) at the 3′-UTR (blue lines) of protein-coding genes (green line), pseudogenes (pink lines), and 3′-UTR and/or gene body of lncRNAs, miRNAs may competitively bind to these MREs. Binding with the pseudogene or lncRNA sequesters the miRNA, preventing miRNA from interacting with the mRNA and altering the level of mRNA compared to that obtained in the absence of or with reduced levels of the pseudogenes or lncRNAs. A microRNA can bind to many mRNAs. Thus, a microRNA binding to an mRNA, for example, mRNA1, may also sequester the miRNA, preventing binding to the other mRNA targets. A pseudogene, lncRNA, and mRNA may, thus, act as “ceRNA” [131,132].

MicroRNAs are generally negative regulators of protein-coding genes (PCGs) by interacting with miRNA response elements (MREs) at 3′-UTR of the gene. MREs are present at the PCG’s exons or, sometimes, at UTRs. MRE–miRNA interactions mostly decrease the stability of the target mRNAs or, in some cases, interfere with the translation. Many publications show that lncRNAs interact with mature miRNAs in animals and plants. The fate of the miRNA or lncRNA in miRNA–lncRNA interaction is not well-established. In animal systems, the direct or indirect destabilization of both miRNA and lncRNA has been reported [124]. Nevertheless, it is unclear in plants. However, miRNA–lncRNA interactions have been shown to sequester the miRNA from binding to its targets in animals [125]. Several predicted and few experimental studies in plants have shown that miRNA is requested due to the interactions with lncRNA in different plants with diverse conditions, and reviewed [126,127,128,129]. A database (PeTMbase) for finding such interactions is available [130]. LncRNAs that interact with miRNAs are known as competing endogenous RNAs (ceRNAs), “sponges,” or endogenous target mimics (eTMs), and can regulate the expression of target genes of miRNAs. Based on several experimental results in animals, Salmena L et al. [131] hypothesized that, “in addition to the conventional microRNA→RNA function, a reversed RNA→microRNA logic exists in which bona fide coding and non-coding RNA targets can crosstalk through their ability to compete for microRNA binding”, commonly referred to as the “competing endogenous RNA” (ceRNA) hypothesis (Figure 12). According to this theory, microRNA response elements (MREs) actively interact with one another to control the levels of their respective expression [131].

**Figure 13:**
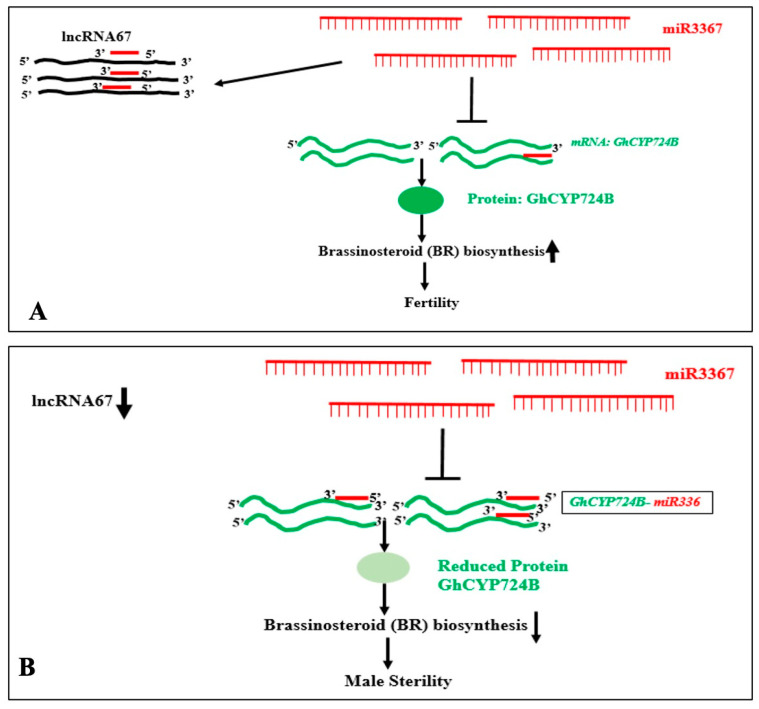
Interactions of microRNA (miR3367), its target protein-coding gene (GhCYP724B), and lncRNA (lncRNA67) in *Gossypium hirsutum* (cotton). (**A**) The interaction of miR3367 with the lncRNA67 sequesters the miRNA and prevents its interaction with its target GhCYP724B in fertile cotton (*G. hirsutum*) line 2074B. High levels of GhCYP724B in cooperation with other proteins, enhance Brassinosteroid (BR) biosynthesis and maintain fertility. (**B**) In the cytoplasmic male sterile line 2074A, in the absence of or with reduced levels of lncRNA67, miR3367 can interact with the target GhCYP724B mRNA, suppressing the expression and reducing the GhCYP724B protein. This reduces the BR biosynthesis, resulting in male sterility [127].

In Arabidopsis, the expression of miR399 and lncRNA IPS1 is enhanced by phosphate starvation. PHO2 mRNA is a target of miR-399. The miR-399 can also interact with the IPS1 RNA, which sequesters miR-399, preventing its interaction with PHO2 mRNA, the target of miR-399, which accumulates more when IPS1 is overexpressed. When miRNA targets PHO2, IPS1 serves as a decoy [133]. In sea buckthorn fruits, two microRNAs, namely, miR156a and miR828a, can interact with cytoplasm-enriched lncRNAs LNC1 and LNC2. It has been demonstrated that miR156a and miR828a decrease SPL9 and promote their respective MYB114 targets. Anthocyanin levels are modulated by such interactions. By functioning as eTMs of miR156a and miR828a, LNC1 and LNC2 may control the expression of SPL9 and MYB114 [129]. In barley, 32 lncRNAs could act as endogenous target mimics (eTMs), potentially decoying the transcriptional suppression activity of 18 miRNAs. For example, interactions of lncRNAs like TCONS_00051546-miR1130, TCONS_00004839-miR159a, (TCONS_00022441, TCONS_00045158)-miR6191, and others were predicted. The findings imply that lncRNA expression may control the Boron-stress response through the cooperative interaction of target transcript modules that code for miRNA and eTM [134]. FRILAIR can modify the expression of LAC11a during the ripening process of strawberry fruit by functioning as a noncanonical target mimic of miR397 [115]. In *Malus spectabilis* (Asiatic apple), miRNA858 targets three lncRNA: MSTRG8246.1 (eTM858-1), MSTRG24337.2 (eTM858-2), and MSTRG.33331.1 (eTM858-3). It has been confirmed that miRNA858 targets the MsMYB62-like gene. Apple color is controlled by MsMYB62-like, a negative regulator of anthocyanin biosynthesis that may also control the anthocyanin biosynthesis gene MsF3′H under low-nitrogen (LN) environments. The expression of MsMYB62-like decreased in the LN growth condition. Additionally, this study demonstrates that eTM858-1 and eTM858-2 are endogenous miR858 target mimics [135]. The interaction of miR3367 with the lncRNA67 sequesters the miRNA and prevents its interaction with its target GhCYP724B in fertile cotton (*Gossypium hirsutum*) line 2074B. High levels of GhCYP724B in cooperation with other proteins enhance Brassinosteroid (BR) biosynthesis and maintain fertility. In the cytoplasmic male sterile line 2074A, in the absence of or with reduced levels of lncRNA67, miR3367 can interact with the target GhCYP724B mRNA, suppressing the expression and reducing the GhCYP724B protein. This reduces the BR biosynthesis, resulting in male sterility [127]. This result is shown in Figure 13.

The competitive endogenous RNA (ceRNA) concept argues that lncRNAs can sponge and inactivate miRNAs, ultimately influencing mRNA targets of the miRNA by altering the degradation or silencing the mRNA translation, therefore affecting the levels of protein-coding genes [131]. The target PCG levels increase, and the miRNA levels fall as a result of lncRNA–miRNA interactions. Therefore, the ceRNA hypothesis also suggests that there is a negative correlation between the expression levels of lncRNA and miRNA and a positive correlation between the expression of mRNA [124].

### 4.2. Scaffold lncRNAs

In plants, lncRNAs can function as scaffolds or adaptors, bringing two or more proteins into a complex, like ribonucleoprotein (RNP) complexes, chromatin-modifying proteins mainly at the chromatin. LncRNA might thus operate as a “central platform” where various related transcription factors, transcription repressors, chromatin-modifying proteins, etc. collaborate at the regulatory regions, affecting the expression of neighboring genes.

**Figure 14:**
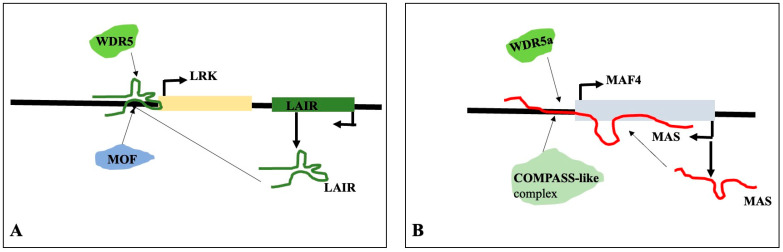
LncRNAs LRK Antisense Intergenic RNA (LAIR) (**A**) and MAS, an antisense of MAF4 (**B**), can act as scaffolds to regulate the expression of LRK and MAF4, respectively. LAIR binds directly to the genomic regions of LRK1 and recruits histone-modifying proteins such as MOF and WDR5. MAS can bind to the genomic MAF4 and recruits WDR5 and COMPASS-like complexes to activate the expression of MAF4.

The antisense strand of the adjacent gene LRK (leucine-rich repeat receptor kinase) is transcribed as the natural antisense lncRNA LAIR (LRK Antisense Intergenic RNA). LAIR overexpression increases the rice grain yield and LRK1 gene expression. The activated LRK1 genomic region is enriched with H3K4me3 and H4K16ac, which are linked to active gene transcription, as is evident from the chromatin immunoprecipitation assay. LAIR helps in recruiting histone-modifying proteins like MOF and WDR5 by directly binding to the genomic regions of LRK1. Therefore, in order to control the gene expression and rice grain yield, LAIR serves as a platform for the recruitment of MOF and WDR5 to the genomic region of LRK [136]. It has been demonstrated that the lncRNA MAS, cold-induced natural antisense of MADS AFFECTING FLOWERING4 (MAF4), regulates MAF4 expression and suppresses blooming. The chromatin immunoprecipitation results reveal that MAS can accumulate the H3K4me3 marker at the MAF4 locus and the expression of MAF4. The activation of MAF4 by MAS requires the interaction of MAS with the genomic region of MAF4 and the recruitment of WDR5a, the core component of the COMPASS-like complex that contains ASHL1, ASHL2, and other proteins. The recruitment of MAS and WDR5a, together with the COMPASS-like complexes, enhances H3K4me3 and the expression of MAF4 [137]. The result is summarized in Figure 14.

#### LncRNA—DNA/Chromatin Interactions

**Figure 15:**
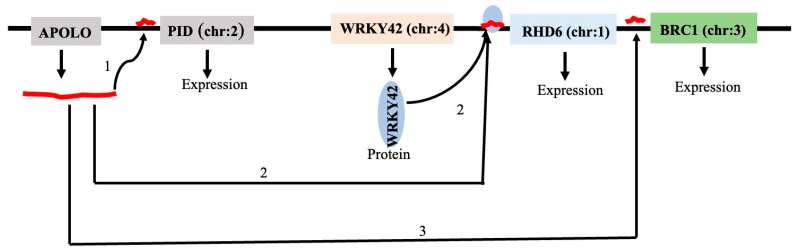
Transcription regulation of different protein-coding genes by APOLO in cis (PID), and WAG2, homologs of PID in trans. PID and WAG2 encode two kinases in charge of determining the position of PIN auxin transporters in the cell membrane, thus modulating auxin efflux [123], and RHD6 and BRC1 in trans by interacting with DNA/chromatin. See references 1: [5,123]; 2: [138], and 3: [140]. For details, see the text.

It has been demonstrated that the lncRNA AUXIN-REGULATED PROMOTER LOOP (APOLO) fine-tunes its transcriptional activity by dynamically regulating the creation of a chromatin loop in cis between its locus and its neighboring gene PID. RNA interference (RNAi) increased the development of a local chromatin loop by knocking down APOLO. APOLO is co-expressed with other auxin-responsive genes and is activated by auxin [5]. Through sequence complementarity and the creation of DNA–RNA duplexes, or R-loops, the lncRNA APOLO interacts with several physically separate genes. The interaction of the APOLO alters the three-dimensional conformation of its target regions by decoying the polycomb repressive complex 1 (PRC1) component LHP1 and regulating the transcription of the target genes. In total, 1974 putative APOLO targets in wild-type *A. thaliana* were found by chromatin isolation using APOLO-RNA purification and DNA sequencing; 2468 differentially expressed genes were identified by comparing the gene expression of the wild-type and 35S: APOLO (highly expressing APOLO) plants; and 2468 differentially expressed genes were identified by comparing the gene expression of the wild-type and 35S: APOLO (highly expressing APOLO) plants. Comparing the potential targets of APOLO and genes that were deregulated in the presence of high levels of APOLO, 187 genes were identified. These genes could be direct targets of APOLO [123]. The analysis further reveals that APOLO could target 17 genes coding for Extensins (EXTs), namely, EXT3, EXT6, EXT8, EXT9, EXT10, EXT12, EXT15, EXT17, and EXT18, and EXT-related proteins like RHD6, LRX2, FLA7, PIP5K3, etc. (Supplementary Table S1 of the published paper [138] involved in root hair (RH) growth and expansion. Cell wall remodeling molecules, including EXTs and EXT-related proteins, are critical for the above processes. The overexpression of the lncRNA APOLO directly or indirectly increases the expression of many genes like EXTs (Supplementary Table S2 of the published paper [138]). Among them, APOLO directly regulates the expression of transcription factor (TF) RHD6 and indirectly regulates RSL2 and RSL4. These TFs play an important role in RH development. APOLO is further observed directly interacting with transcription factor WRKY42 at the protein level. RHD6 cannot be activated by low temperatures without WRKY42. The outcome demonstrates that polycomb-dependent H3K27me3 dynamic deposition is necessary for the control of the RHD6 expression in response to the cold. The outcome demonstrates that polycomb-dependent H3K27me3 dynamic deposition is necessary for the control of RHD6 expression in response to the cold. At low temperatures, the complex WRKY42–APOLO stimulates transcription, modifies the epigenetic environment of RHD6, and promotes root hair (RH) development. WRKY42 interacts with the RHD6 promoter and alters the expression of RHD6. RHD6 activation also causes the production of RSL2 and RSL4, which regulate the transcriptional RH program and cause cell expansion in response to the cold [138,139]. This result is summarized in Figure 15.

It has been demonstrated that the rice enhancer of the zeste gene OsiEZ1 interacts with the lncRNA RICE FLOWERING ASSOCIATED (RIFLA), which is found in the first intron of the gene OsMADS56, to control the expression of the host gene OsMADS56. The Arabidopsis histone H3K27-specific methyltransferase genes SWINGER (SWN) and CURLY LEAF (CLF) are homologs of OsiEZ1. Under normal circumstances, the OsMADS56 expression was comparatively high, whereas the OsiEZ1 and RIFLA expression were low. The OsMADS56 expression was elevated in plants carrying the osiez1 mutation. The RIFLA expression was downregulated in this situation. Additionally, it was noted that RIFLA and OsiEZ1 formed a complex. These findings demonstrate that RIFLA and OsiEZ1 epigenetically regulate the floral repressor activity of OsMADS56 [20]. The morphological and physiological reactions that enable plants to escape being shadowed by nearby plants are the hallmarks of shade avoidance syndrome (SAS). The SAS responses are triggered by the decrease in the ratio of red (R) to far-red (FR) radiation (R/FR ratio), which is caused by the absorption of R and the reflection of FR photons by tissues that contain chlorophyll. Auxins regulate the SAS response. Additionally, it has been demonstrated that auxin induces APOLO [5]. By controlling the transcription of BRANCHED1 (BRC1), a master regulator of shoot branching in Arabidopsis, through chromatin looping driven by variations in light exposure, a low R/FR ratio regulates APOLO and contributes to SAS. This suggests that APOLO plays a role in SAS. Additionally, by coordinating the actions of LHP1 and VIM1 on histone and DNA methylation, APOLO may contribute to leaf hyponasty through the epigenetic control of the auxin-synthesis-related gene YUCCA2/YUC2 on chromosome 4 [100], and the PID [5] and PID homolog WAG2 on chromosome 3 of *A. thaliana* [123], two genes that encode kinases involved in auxin redistribution and PIN transporter phosphorylation at low R/FR ratios [140]. In conclusion, by binding with DNA to create the R-loop (DNA-RNA) and modifying the transcription of the cis target genes (PID), trans target genes (RHD6, BRC1, YUCCA2/YUC2, and WAG2), and other genes in *A. thaliana*, APOLO can modify the local three-dimensional chromatin conformation. SAS and RH development are affected by the changed expression of these genes by APOLO. The expression of the target genes is controlled by the interactions of RIFLA with the protein OsiEZ1 and APOLO with the protein WRKY42.

**Figure 16:**
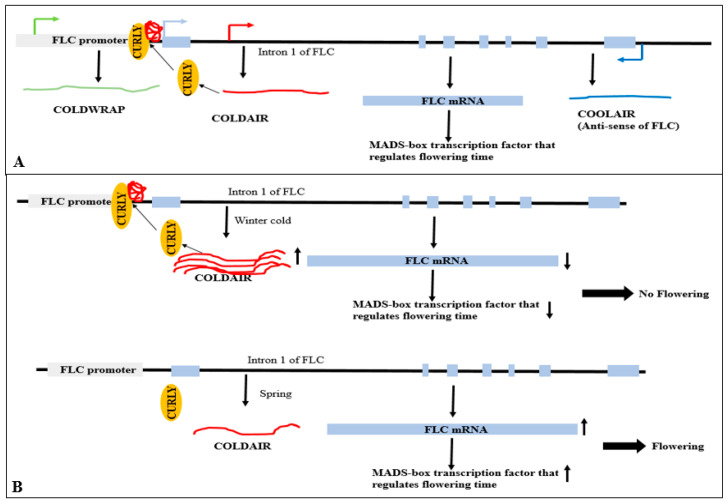
(**A**): On chromosome 5 of *Arabidopsis thaliana*, the lncRNAs COLDAIR, COLDWRAP, and COOLAIR are arranged around the FLOWERING LOCUS C (FLC) gene, which codes for the MADS-box transcription factor. The transcription start sites are indicated by horizontal lines with arrowheads; the colors of the arrowheads and the transcripts are identical. An enzyme component of PRC2 (shown by filled ovals) and a homolog of mammalian EZH2, CURLY, interacts with COLDAIR and directs CURLY to FLC. (**B**): The expression of COLDAIR rises in response to cold (winter), which inhibits flowering by reducing the expression of FLC.

The floral repressor FLOWERING LOCUS C (FLC), which encodes the MADS-box transcription factor, is epigenetically stably repressed in Arabidopsis due to the tri-methylated histone H3 Lys 27 being enhanced by the winter cold. Flowering is negatively regulated and repressed downstream by FLC. By adjusting the flowering time in response to extended low temperatures, FLC participates in the vernalization pathway. Polycomb repressive complex 2 (PRC2) is an evolutionarily conserved repressive complex that mediates epigenetic modification in FLC. It has been demonstrated that the vernalization-mediated epigenetic suppression of FLC requires the long non-coding RNA COLDAIR (Cold Assisted Intronic non-coding RNA). It has been demonstrated that COLDAIR binds to CURLY, an enzymatic component of PRC2 and a homolog of mammalian EZH2, and directs CURLY/PRC2 to FLC [141]. The expression of FLC and blooming plants is further regulated by two additional lncRNAs that code from the FLC promoter in the sense and antisense of FLC, respectively, and these are called COLDAIR and COOLAIR [17,142] (Figure 16).

SVALKA is a natural antisense RNA. It plays a crucial role in controlling how *A. thaliana* reacts to low temperatures. On chromosome 4, SVALKA is transcribed both proximally and in an antisense direction to the genes CBF1 and CBF3/DREB1A it targets. SVALKA has two main isoforms: SVK-S (696 nucleotides), predominantly expressed at 22 °C, and SVK-L (2102 nucleotides), predominantly expressed at 4 °C. Nascent SVK-L RNAs interact with CBF1 mRNA to produce a double-stranded lncRNA–mRNA complex, which DICER-LIKE (DCL) proteins recognize as a substrate. Short dsRNA fragments are produced when DCL recognizes this dsRNA substrate. HUA ENHANCER 1 (HEN1) stabilizes these pieces by methylation. The RNA-induced silencing Complex (RISC) is formed when one of the guide strands of the dsRNA fragment is loaded onto ARGONAUTE1 AGO1. Instead of totally suppressing the expression of CBF1, SVK-L serves to maintain its balance. The CBF1 sense RNA’s half-life is shortened, but its transcription rate stays constant. To adjust the transcriptional response to cold temperatures, SVALKA uses the above three approaches.

At 4 °C, isoform SVK-S is the predominant isoform. Antisense RNAPII may collide with sense RNAPII, resulting in premature CBF1 transcription termination. Following the collision event, both the premature CBF1 mRNA and SKV-S transcripts are degraded by a HEN2/exosome-mediated mechanism. SVK-S may also negatively affect the expression of CBF1 in response to cold temperatures. After being exposed to the cold, SVK-S expression disruption increases CBF1 expression. CBF1 expression is downregulated when SVK is overexpressed. Additionally, SVK-S has the ability to control CBF3 levels throughout the cold response for extended periods of time after the exposure to low conditions. PRC2/CURLY LEAF (CLF) can be recruited by SVK-S to the CBF3 gene’s coding region. PRC2’s CLF methyltransferase subunit interacts with SVK-S. PRC2 suppresses the expression of the CBF3 gene through promoting the deposition of the restrictive histone mark H3K27me3. Therefore, by causing the epigenetic silencing of CBF3 and controlling CBF1 transcript levels following the initial induction, SVALKA adversely regulates both CBF1 and CBF3 [143].

### 4.3. Guide lncRNA

**Figure 17:**
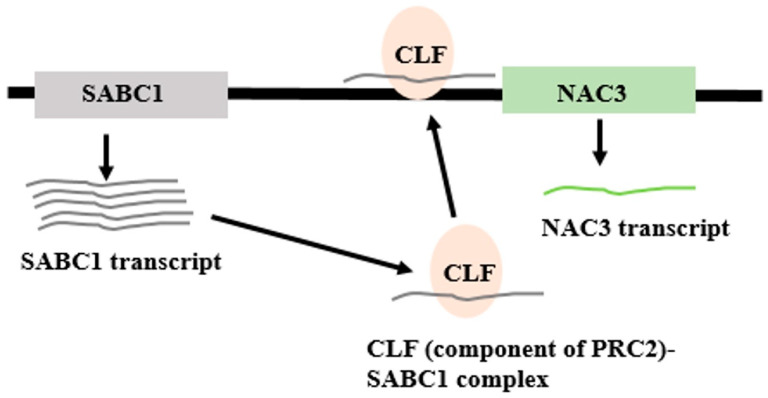
Cartoon presentation of lncRNA SABC1 to guide the recruitment of the PRC2 complex to a regulatory region. When CLF, a member of the repressive PRC2 complex, interacts with lncRNA SABC1, it directs CLF to recruit to the regulatory domain of the nearby transcription factor NAC3, which represses NAC3 production in healthy plants. SABC1 expression is decreased, and PRC2 complex recruitment is inhibited in plants infected with pathogens (*P. syringae* or TuMV) to inhibit the transcription of NAC3 and its target to synthesize salicylic acid, the infection-preventive agent [144].

The lncRNA salicylic acid biogenesis controller 1 (SABC1), an infection-responsive lncRNA, has been identified to fine-tune salicylic acid biosynthesis, which, in turn, balances plant immunity and growth. SABC1 recruits the polycomb repressive complex 2 (PRC2) via interacting with CURLY LEAF (CLF), an important component of PRC2 and responsible for catalyzing H3K27me3 to its nearby gene NAC3. A transcription factor is encoded by NAC3. Isochorismate synthase 1 (ICS1), a crucial enzyme that catalyzes the production of salicylic acid, is transcriptionally activated by NAC3. Thus, via inhibiting NAC3 and ICS1 transcriptions, SABC1 suppresses the synthesis of salicylic acid and plant immunity. SABC1 is downregulated during pathogen infection to increase plant tolerance to viruses and bacteria [144]. The result is shown in Figure 17.

### 4.4. Signal lncRNA

LncRNA can act as a signaling molecule by interacting mainly with proteins or mRNAs other than transcription factors/repressors/regulators. The ABA degradation pathway has been linked to the possible functions of mRNA–lncRNA interactions in pear; lncRNA–microRNA may also play a part [145]. The functional analysis of differentially expressed lncRNAs in response to the cold identifies a trans-acting lncRNA CRIR1, which interacts with MeCSP, a cold shock domain protein, to improve the translation efficiency at low temperatures in cassava. Such an interaction can confer cold stress tolerance. It has further been shown that the increased expression of many cold-related genes in CRIR1-over-expressing plants during cold stress was independent of transcription regulation by CRIR1 [146].

## 5. Stress-Induced lncRNA

Plants constantly face a variety of environmental stresses that can be broadly classified as biotic and abiotic. These stresses have significant effects on the growth, development, and production of plants. While biotic stresses involve living organisms like bacteria, fungi, viruses, nematodes, and insect pests, abiotic stresses—such as drought, salinity, temperature extremes, heavy metal toxicity, and nutritional deficiencies—are caused by non-living environmental variables. To cope with these challenges, plants reprogram their transcriptomes extensively, resulting in the dynamic modulation of numerous genes, including lncRNAs. Numerous plant species have been identified to have stress-responsive lncRNAs as a result of the developments in next-generation sequencing and bioinformatics techniques. Despite the rapid progress, the functional characterization of these lncRNAs remains limited, and their regulatory networks are largely predicted based on expression patterns or computational analyses. In addition to offering important insights into plant biology, an understanding of the dynamic and complex roles of lncRNAs in the plant stress response holds promise for improving agricultural resilience to adverse environmental conditions and global climate change. This section briefly reviews the current knowledge on plant lncRNA responses to various abiotic and biotic stresses, summarizing the recent findings and highlighting their regulatory roles and potential agricultural applications.

### 5.1. Long Non-coding RNAs in Response to Abiotic Stress

**Figure 18:**
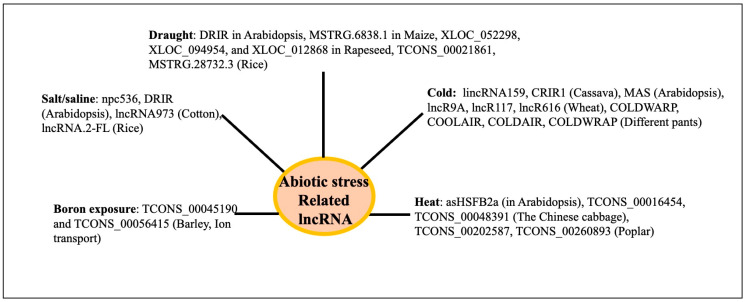
LncRNA in different abiotic stresses. Details with references are shown in Supplementary Text S2.

As briefly mentioned above, plant lncRNAs play a role in the growth and development of several organs, including the root, leaf, seed, endosperm, and seed germination, among others. Furthermore, lncRNAs have recently been evaluated and linked to environmental stressors such as drought, salt, flood, heat, and cold [25,28,110,111] [Supplementary Table S1]. Common crops, which are divided into (a) grain crops like wheat, corn, and rice, (b) oil crops like soybean, peanut, and rapeseed, (c) sugar crops like sugarcane and beet, (d) fiber crops like cotton and hemp, (e) beverage crops like tea and coffee, and (f) vegetables like tomatoes, have been reviewed to show that LncRNAs play a significant role in response to a variety of abiotic stresses [119]. In most of these studies, different types of abiotic stresses deregulated thousands of lncRNAs [147,148,149,150,151,152,153,154,155,156,157,158,159]; however, the exact role of these lncRNAs has been deciphered in a limited number of cases. The involvement of representative lncRNAs in different plants is summarized in Figure 18, and the details are shown in Supplementary Text S2.

### 5.2. Role of lncRNA in Biotic Stress/Plant–Pathogen Interactions Mediated Through lncRNA

Numerous pathogens, such as bacteria, fungi, viruses, nematodes, and insect pests, provide a constant threat to plants. Plants have developed complex defense systems requiring dynamic transcriptional reprogramming to deal with various biotic stressors. Among these responses, lncRNAs are emerging as key regulatory molecules for modulating plant immunity. LncRNAs exert their influence through diverse modes of action, such as acting as miRNA sponges, precursors of small RNAs, epigenetic regulators, or components of competing endogenous RNA (ceRNA) networks. Recent studies using high-throughput sequencing and functional genomics have identified numerous lncRNAs that are differentially expressed upon pathogen infection, revealing their critical roles in regulating plant defense responses [Supplementary Table S2]. Supplementary Table S2 depicts a diverse array of lncRNAs associated with plant disease responses, highlighting their roles in modulating immune signaling and pathogen defense mechanisms. The following sections summarize notable examples of pathogen-specific lncRNA responses across various plant species.

#### 5.2.1. Fungal Pathogens

Twenty-three lncRNAs in wheat displayed reactivity to both heat stress and powdery mildew infection (named TalnRNA), whereas 48 lncRNAs solely responded to powdery mildew infection (designated TapmlnRNA). Following an infection with powdery mildew, there was an upregulation of both MiR2004 and its host lncRNA genes TapmlnRNA19 and TalnRNA5. In young spike tissues, TapmlnRNA19 accumulated more selectively. Although talnRNA5 was expressed in all tissues, the seeds had comparatively greater expression levels. LncRNA TalnRNA5 and TapmlnRNA19 both host miR2004, although they showed different expression patterns. In addition, another lncRNA TapmlnRNA8 (host gene for miR2066) demonstrated specificity for infections caused by powdery mildew [157]. After being infected with *Fusarium oxysporum*, the expression of several natural antisense lncRNAs in *Arabidopsis* changed. It is likely that pathogen infection co-induces these antisense lncRNAs and their sense transcripts [160].

#### 5.2.2. Viral Pathogens

The lncRNAs slylnc0195 and slylnc1077 in tomato functioned as “decoys” for miR166 and miR399, respectively, during the infection caused by the tomato yellow leaf curl virus (TYLCV) [161]. In the case of the resistance of *Paulownia tomentosa* to phytoplasma infection, 23 lncRNAs served as target mimics that interacted with 33 miRNAs. A ceRNA network consisting of five lncRNAs, five miRNAs, and fifteen mRNAs shed light on the progression of Paulownia witches’ broom disease [162]. Comparative analyses of transcriptomes from resistant and susceptible tomato plants infected with *Phytophthora infestans* revealed several lncRNA genes that participate in ceRNA networks influencing resistance through interactions with miRNAs and mRNAs. Notable modules included the lncRNA42705-miR159-MYB and lncRNA40787-miR394-JA biosynthesis pathways, enhancing resistance [163,164,165]. Silencing lncRNA10865 aggravated Sugarcane mosaic virus (SCMV) symptoms, while silencing lncRNA14234 alleviated SCMV symptoms in infected Maiz [166]. Rice affected by the Rice Black-Streaked Dwarf Virus exhibited 344 differentially expressed lncRNAs, while rice infected with the Rice Stripe Virus displayed 176 differentially expressed lncRNAs. A subset of 21 lncRNAs commonly responded to both viruses, impacting transcriptional regulation and hormone signaling pathways [167]. In maize infected by the Sugarcane mosaic virus (SCMV), several lncRNA–miRNA–mRNA regulatory modules were identified, such as lncRNA10865-miR166j-3p-HDZ25/69, influencing susceptibility and resistance outcomes [166].

#### 5.2.3. Bacterial Pathogens

In rice, leaves affected by the bacterium *Xanthomonas oryzae* pv. oryzae (Xoo) result in a significant blight, with 73 lncRNAs showing deregulation. It has been noted that 39 protein-coding genes related to jasmonate (JA) engage with the deregulated lncRNAs. The increased levels of ALEX1 improve resistance against Xoo and stimulate JA signaling [168]. Transcriptomic studies reveal complex interactions between plants and bacteria regulated by long non-coding RNAs (lncRNAs). Rice infected with *Xanthomonas oryzae* shows enhanced resistance through lncRNAs like ALEX1, which activate jasmonic acid signaling. LncRNAs also encode antimicrobial peptides (AMPs) critical for the defense against pathogens like *Rhizoctonia bataticola*. In legumes, lncRNAs (GmENOD and mtENOD40) facilitate nodulation and symbiosis by interacting with RNA-binding proteins (RBPs) and encoding regulatory peptides. In *Arabidopsis*, the long non-coding RNA Salicylic Acid Biogenesis Controller 1 (SABC1) responds to pathogen infections and regulates the production of salicylic acid (SA), thus balancing plant immunity and growth. SABC1 interacts with CURLY LEAF (CLF), an essential part of the polycomb repressive complex 2 (PRC2), facilitating the modification of H3K27me3. SABC1 engages CLF-PRC2 to inhibit the expression of the NAC transcription factor gene through H3K27me3 modification. In normal circumstances, SABC1 lowers SA production by decreasing the levels of NAC3 and isochorismate synthase 1, which is a crucial enzyme in the biosynthesis of SA. Upon infection with bacterial pathogen *Pseudomonas syringae* or viral pathogen TuMV, the SABC1 expression is downregulated, relieving repression and enhancing immune responses [144].

#### 5.2.4. Nematode Pathogens

In peanut infected by root-knot nematodes, approximately 4450 differentially expressed lncRNAs were identified, some potentially regulating oxidation–reduction processes [169]. In soybeans affected by the cyst nematode (*Heterodera glycines*) and *Rotylenchulus reniformis*, a total of 384 and 283 potential lncRNAs were discovered, respectively. Numerous lncRNAs exhibited comparable expression patterns, indicating shared nematode-response mechanisms that involve salicylic acid and jasmonic acid signaling, along with plant hypersensitive reactions [170]. In tomato, the differential expression of lncRNAs during *Meloidogyne incognita* infection highlighted distinct early- and late-stage responses. Several lncRNAs functioned as miRNA sponges or encoded peptides/microproteins, enhancing the resistance to root-knot nematodes [171].

#### 5.2.5. Insect Pest Interactions

A transcriptomic analysis of cotton infested by whitefly revealed 606 differentially expressed lncRNAs. The co-expression network construction identified lncA07 and lncD09 as regulatory hubs influencing jasmonic acid pathways and plant resistance. Knock-out mutants demonstrated a reduced resistance to insect pests [172]. A specialized database, PotatoBSLnc (https://www.sdklab-biophysics-dzu.net/PotatoBSLnc, accessed on 2 March 2025), focusing on significant abiotic stresses in potato (*Solanum tuberosum* L.), includes information gathered from 364 RNA sequencing related to 12 biotic stresses across 26 cultivars and wild potato species. This database serves as a valuable resource for studying biotic stresses in potatoes [173].

### 5.3. Immune Response Mediated Through Plant lncRNAs

To combat biotic stresses, plants have evolved complex immune systems that identify these stressors and generate suitable signals to control their growth and development. As a reaction to biotic stress, plants employ immune receptors found on the cell surface as well as those located inside the cells to detect microbial signals and trigger immune responses, such as calcium influx, bursts of reactive oxygen species (ROS), and the activation of mitogen-activated protein kinases (MAPKs). These initial immune responses lead to subsequent transcriptional changes in defense-related genes. The genes associated with defense comprise transcription factors and those involved in hormone production, contributing to late immune responses. While numerous studies have highlighted the significant roles of lncRNAs in the growth, development, and immune responses of animals, few studies are addressing the role of lncRNAs in plant immunity. Numerous studies indicate that a significant number of lncRNAs are activated by pathogens or insect invasions [174], and plants utilize lncRNAs to control their immune responses. The expression levels of lncRNAs vary, either increasing or decreasing, when faced with biotic stress, and this has been documented in a range of plant species. For instance, during the infection of Arabidopsis by *F. oxysporum*, the expression levels of various natural antisense and intergenic lncRNAs were modified. In tomatoes, infection with the Tomato Yellow Leaf Curl Virus (TYLCV) changed the expression levels of numerous lncRNAs, leading to an increase in the expression of slylnc0048, slylnc0049, slylnc0483, slylnc0531, and slylnc0934, while the expression of slylnc0476, slylnc0475, slylnc0673, and slylnc1052 decreased. The overexpression and suppression of lncRNA33732 in tomatoes revealed that lncRNA acts as a positive regulator for tomato resistance against infections by promoting the expression of respiratory burst oxidase (RBOH) and the buildup of H2O2 [175]. Altered levels of approximately 40 lncRNAs have been detected in *Arabidopsis*, Cotton, Peanut, Potato, Rice, Soybean, Tobacco, and Tomato when infected by 14 different pathogens (including viruses, bacteria, fungi, oomycetes, nematodes, and insects) that are probably associated with plant immune responses [176]. A representative result is shown in Table 5.

## 6. Conclusions and Future Perspectives

The development of advanced sequencing technologies and powerful computational tools has enabled the identification and prediction of thousands of lncRNAs across many plant species; many plant species are still left behind. Our understanding of the functional roles of plant lncRNAs is still in its early stages, despite significant advancements in their identification and classification. It is difficult to predict the functions of lncRNAs using sequence-based comparisons due to the lower amount of sequence conservation of lncRNAs. The precise molecular mechanisms by which plant lncRNAs exert their functions are not fully elucidated but are rapidly growing. The principles of modes of actions of lncRNAs for performing the functions could be similar to those that have been well-studied in animal systems, including in humans and mice. The role of lncRNA in abiotic stress or biotic stress is beginning to emerge in laboratory-based studies.

In the present review, we provided a brief overview of the diversity of plant lncRNAs and resources for their information in different databases. We highlighted how plant lncRNAs function as signals, scaffolds, decoys, and guides to modulate gene expression by interacting with DNA/chromatin, mRNA, microRNA, and protein. LncRNAs are regulated by transcription factors and epigenetic factors; these factors could also be regulated by lncRNAs. Long non-coding RNAs have been identified as pivotal regulators of transcription, orchestrating developmental processes and mediating stress responses through a diverse array of mechanisms. We also provide examples of the roles of lncRNAs in growth, development, and adaptability to both biotic and abiotic stress. LncRNAs add an extra layer of genetic regulation, interacting with well-known protein and microRNA-based networks. Additionally, the discovery of some lncRNAs encoding micropeptides blurs the distinction between coding and non-coding functions, suggesting that many lncRNAs may have multifunctional roles. Plant lncRNAs, in specific cases, have been observed to regulate abiotic and biotic stress tolerance. Plant height, tillering capacity, root length, grain size, and resistance to diseases and pests are examples of agronomic traits. These traits significantly influence crop yield, quality, and the ability to withstand biotic and abiotic stresses.

Many lncRNAs identified through RNA sequencing have not yet been functionally characterized; the predicted functions rely heavily on bioinformatics approaches, and their confirmation is carried out by a small-scale experimental approach such as co-expression, co-localization, or physical interaction analyses with proteins or mRNAs. Gain-of-function or loss-of-function studies are limited. The experimental validation of these predicted functions remains incomplete, highlighting the importance of future studies. As we advance with future studies, we might encounter numerous exciting opportunities as well as challenges in our research environment. Understanding what lncRNAs actually do needs systematic validation with advanced technologies. Technologies like CRISPR/Cas9 can be applied to precisely delete the lncRNA loci or promoters without disrupting nearby genes, providing a powerful way to link specific lncRNAs to visible traits in plants. When combined with transcriptomics and chromatin profiling, these mutants can help reveal the molecular pathways that lncRNAs influence. Since lncRNA effects are often subtle or context-dependent, studies should span diverse environmental conditions and developmental stages. Recent CRISPR interference (CRISPRi) approaches explicitly designed for knocking down lncRNAs could be particularly powerful for functional studies. Exploring the mechanisms of functional lncRNAs through mapping with DNA, RNA, and proteins using techniques like Chromatin Isolation by RNA Purification (ChIRP), RNA antisense purification (RAP), and RNA pull-down assays is essential. Understanding whether a lncRNA directs proteins, sponges miRNAs, or serves as a scaffold is also crucial, as is studying its structure to gain insights into its interactions. Future research might explore lncRNA-mediated regulatory networks and their connection to hormone signaling pathways. This is especially relevant when examining the role of lncRNAs in regulating these key agronomic traits and affecting the yield and quality of various model plants [52,88,89,177]. Additionally, environmental stressors such as heat and cold have a substantial impact on plant growth and development, limiting global crop productivity. In summary, further in-depth studies would be necessary before the knowledge we are beginning to gather about the lncRNA in plants can be utilized for the improvement of crops and forests for the improvement in human uses.

## Figures and Tables

**Table 1:** miRNAs in representative plants (miRBase https://www.mirbase.org/, accessed on 2 March 2025).

Species	Common Name	Precursor miRNA	Mature miRNAs
*Arabidopsis thaliana*	Arabidopsis	326	430
*Oryza sativa*	Rice	604	757
*Triticum aestivum*	Wheat	122	125
*Hordeum vulgare*	Barley	69	72
*Zea mays*	Maiz	174	325
*Lotus japonicus*	Wild legume	299	365
*Solanum tuberosum*	Potato	224	343
*Glycine max*	Soybean	684	756
*Brassica rapa*	Mustard	96	157
*Gossypium hirsutum*	Cotton	-	240 *
*Homo sapiens*	Human	1917	2693
*Mus musculus*	Mouse	1234	2013

* http://www.nipgr.ac.in/CoNCRAtlas/, accessed on 2 March 2025.

**Table 2:** Genome size, and protein-coding gene (https://www.ncbi.nlm.nih.gov/datasets/genome/, accessed on 2 March 2025), and lncRNA (https://www.tobaccodb.org/plncdb/, accessed on 2 March 2025) in representative plants.

Species	Common Name	Genome Size	LncRNA	Protein-Coding Gene
*A. thaliana*	Arabidopsis	119 Mb	13,599	27,562
*O. sativa Japonica Group*	Rice	385.7 Mb	11,565	29,427
*T. aestivum*	Wheat	14.6 Gb	43,659	103,787
*H. vulgare*	Barley	4.2 Gb	25,884	31,448
*Z. mays*	Maiz	2.2 Gb	32,397	34,313
*L. japonicus*	Wild legume	553.7 Mb	2936	32,752
*S. tuberosum*	Potato	705.8 Mb	16,485	28,411
*G. max*	Soybean	978.4 Mb	12,577	47,068
*B. rapa*	Mustard	352.8 Mb	17,519	41,403
*G. hirsutum*	Cotton	2.3 Gb	53,721 ^@^	67,584
Dinoflagellate Scrippsiella acuminate ***	Cosmopolitan microalga	~ 51.44 Gb	78,393	116,417 [51]
*H. sapiens*	Human	3.1 Gb	35,934 *	20,078
*M. musculus*	Mouse	2.7 Gb	36,172 **	22,198

* https://www.gencodegenes.org/human/stats_47.html (accessed on 3 February 2025) ** https://www.gencodegenes.org/mouse/stats_M36.html (accessed on 3 February 2025) *** *Scrippsiella acuminata* is a dinoflagellate that forms blooms in the ocean and can cause harmful effects on the environment. It is also known as a cosmopolitan microalga. ^@^ http://www.nipgr.ac.in/CoNCRAtlas/ (accessed on 3 February 2025).

**Table 3:** Representative databases for plant lncRNAs.

Databases	Contain	References
PLncDB	Information on plant lncRNA in 80 species	[58]
CANTATAdb	Catalog computationally predicted 571,688 lncRNAs in 108 plant species; *Papaver somniferum* (opium poppy) had the maximum number of lncRNAs (24,516), followed by *Avena sativa* (Oat), which had 19,158	[59]
GreeNC 2.0	Over 495,000 annotated lncRNAs from 94 plant and algal species are available in this repository	[60]
PlantNATsDB	2,146,803 natural antisense transcripts predicted from 70 plant species are catalogued in PlantNATsDB	[61]
Plant ncRNA database (PNRD)	More than 25,000 ncRNAs from 150 plant species and 11 distinct kinds are present in PNRD	[62]
LncPheDB	In the database, 203,391 known and predicted lncRNA sequences in 9 species are catalogued using a unified reference genome annotation	[63]

**Table 4:** Distribution of plant lncRNAs in subcellular compartments [86].

Plants	Nucleus	Cytoplasm	Ribosome *	Exosome
*A. thaliana*	66.6%	31.8%	0.1%	1.5%
*O. sativa*	53.7%	38.2%	1.1%	7.0%
*Z. mays*	45.9%	44.7%	1.3%	8.1%

* extrapolated.

**Table 5:** Plant lncRNAs associated with immune response.

Stage	Pathogen/Plant	LncRNAs	Target	Activates/ Inhibits Targets
ROS production	Oomycete/tomato	LncRNA33732	RBOH	Activates
	Oomycete/tomato Virus/tobacco	LncRNA16397 LncRNA LMT1	SlGRX AOX-1a	Inhibits Inhibits
Calcium Influx	Abiotic stress/Mulberry	MuLnc1	MuCML27	Inhibits
MAPKs cascades	Oomycete/*Arabidopsis*	nalncFL7	nalncFL7-FL7-HAI1-MAPK3/6	Activates
NLR	Oomycete or water mold/Tomato Oomycete or water mold/Tomato Oomycete or water mold/Tomato	LncRNA23468 Sl-lncRNA15492 LncRNA08489	miR482b miR482a miR482e-3p	Activates Activates Activates
Defense-related genes	Bacteria/*Arabidopsis* Oomycete/Tomato Flagellin (bacterial PAMP)/*Arabidopsis*	ELENA1 LncRNA39026 ASCO	MED19a SlPR1, SlPR2, SlPR3, SlPR5 NSRs	Activates Activates Activates
Modulate defense-related genes				
Genes related to Salicyclic Acid (SA) synthesis	Virus/Tobacco Virus/*Arabidopsis*	LMT1 SABC1	AOX-1a NAC3	Inhibits Activates
Genes related to Jasmonic Acid (JA)synthesis	Bacteria/*O. sativa* Fungus/Cotton Insect/Tobacco Fungus/Cotton	ALEX1 LOX3 JAL1 and JAL3 GhlncNAT-ANX2, GhlncNAT-RLP7	AZ8, MYC2, PR1a, etc. GhLOX3 WIPK, WRKY3, WRKY6, etc. ANX2, RLP7	Activates Activates Activates Inhibits
Genes related to JA and ethylene synthesis	Oomycete/tomato	LncRNA39896	SlHDZ34 SlHDZ45	Inhibits

## Data Availability

The data are included in the review and Supplementary Materials, or referenced in the review. Data sharing is not applicable.

## Supplementary Materials

The following supporting information can be downloaded at: https://www.mdpi.com/article/10.3390/genes16070765/s1. Table S1. Functions of lncRNAs in representative plants. Table S2. Plant disease-related lncRNAs. Supplementary Text S1. Biogenesis and mechanism(s) of actions of microRNA. Supplementary Text S2. Long Noncoding RNAs in response to abiotic stress. References [178,179,180,181,182,183,184,185,186,187,188,189,190,191,192,193,194,195,196,197,198,199,200,201,202,203,204,205,206,207,208,209,210,211,212,213,214,215,216,217,218,219,220,221,222,223,224,225] are cited in the Supplementary Materials.

## Abbreviations

The following abbreviations are used in this manuscript:

ncRNA	non-coding RNA
PCG	protein-coding genes
lncRNA	long non-coding RNA
miRNA	microRNA
CircRNA	circular RNA
rRNAs	ribosomal RNAs
tRNAs	transfer RNAs
snRNA	small nuclear RNA
SnoRNA	small nucleolar RNA
siRNAs	small interfering RNAs
tRFs	tRNA-derived small RNA fragments
phasiRNA	phased siRNA
tasiRNA	trans-acting siRNAs
easiRNA	epigenetically activated siRNAs
scaRNAs	small-Cajal-body-associated RNAs
PmiREN	Plant miRNA Encyclopedia
RISC	RNA-induced silencing complex
RdDM	RNA-directed DNA methylation
shRNAs	short hairpin RNAs
PLncDB	Plant Long non-coding RNA Database
GreeNC, v2.0	Green Non-Coding Database
PNRD	Plant ncRNA database
RDR2	RNA-directed RNA polymerase 2
FRILAIR	fruit-ripening-related long intergenic RNA
ceRNAs	competing endogenous RNAs
LAIR	LRK Antisense Intergenic RNA
APOLO	AUXIN-REGULATED PROMOTER LOOP
TYLCV	tomato yellow leaf curl virus
SABC1	Salicylic Acid Biogenesis Controller 1
ROS	reactive oxygen species; MAPK- mitogen-activated protein kinase
FLC	FLOWERING LOCUS C
COLDWRAP	cold-of-winter-induced non-coding RNA from the promoter
COLDAIR	Cold Assisted Intronic non-coding RNA
LDMAR	long-day-specific male-fertility-associated RNA
ASCO	alternate splicing competitor long non-coding RNA
IPS1	INDUCED BY PHOSPHATE STARVATION 1
HID1	HIDDEN TREASURE 1
ASL	ANTISENSE LONG
PILNCR1	Pi-deficiency-induced long-non-coding RNA1
GARRs	GIBBERELLIN-RESPONSIVE lncRNAs
TRABA	Trans-acting of BGLU24 by lncRNA
ELENA1	ELF18-INDUCED LONG-NON-CODING RNA1
ChIRP	Chromatin Isolation by RNA Purification
