# Supplementary material for: LncRNAOmics: A Comprehensive Review of Long Non-Coding RNAs in Plants
Source: Genes (Basel). 2025 Jun 29;16(7):765. doi: 10.3390/genes16070765 (PMC12294436; doi:10.3390/genes16070765)
Supplement: Supplementary file 1 [file genes-16-00765-s001.zip › Supplementary Table S1.pdf]

Supplementary Table S1: Functions of lncRNAs in representative plants

**LncRNA (size)**	**Genomic loci/ type of lncRNA**	**Plants**	**Interacting partners**	**Mechanism of actions**	**Molecular Functions**	**Biological function**	**Reference**
Development and growth (Organogenesis, Seed growth and germination, flowering time, photomorphogenesis, leaf development, vernalization, etc.							
ENOD40	Antisense lncRNA	*Medicago truncatula*	MtRBP1, a homolog of nuclear speckle RNA binding protein (RBP)	ENOD40 re-localizes MtRBP1 to the cytoplasm from the nucleus speckle	Sequestration of nuclear protein in the cytoplasm	Root nodule organogenesis	[70,121,122]
HID1 (nc3020)	HID1 (nc3020) localized in a cluster of ncRNAs (3020, 3019, 3018 and 3017)	Arabidopsis	HID1 is assembled into large nuclear protein-RNA complex(es) (around 500 kDa) and is associated with the chromatin of the first intron of PIF3.	HID1 represses phytochrome-interacting factor 3 (PIF3) on chromosome 1.	Transcription repression by interacting with DNA/chromatin	Seedling photomorphogenesis	[199]
asDOG1	The 3′ regions of the Delay of Germination 1 (DOG1) gene, antisense lncRNA	Arabidopsis	asDOG1 interacts with DOG1	asDOG1 strongly suppresses DOG1 expression during seed maturation in cis	Negative regulation of seed dormancy by asDOG1	Seed dormancy	[18]
APOLO	Intergenic lncRNA	Arabidopsis		Modulates the expression of BRANCHED1 (BRC1), a master regulator of shoot branching in Arabidopsis by chromatin looping	Modulates the expression of BRC1	Shoot branching, Shade avoidance syndrome,	[140]

				influenced by changes in light exposure		characterized by morphological and physiological responses that allow plants to avoid being shaded by neighbors, increase the interception of sunlight in plant canopies	
LNC1 and LNC2		Hippophae L (Sea buckthorn)		Act as endogenous target mimics of miR156a and miR828a to reduce SPL9 and induce MYB114 expression, respectively	Decreased SPL9 and increased MYB114 results in increased and decreased anthocyanin content respectively	Fruit ripening and quality	[129]
RIFLA	The first intron of the OsMADS56 gene, Intronic lncRNA	*Oryza sativa* L (rice)		RIFLA-overexpressing transgenic rice plants flowered earlier than the wild-type	RIFLA formed a complex with OsiEZ1; a homolog of Arabidopsis is required for the inhibition of OsMADS56 expression	Flowering	[20]

COOLAIR	Antisense of FLC	Arabidopsis		COOLAIR is sufficient to confer cold-induced silencing of the reporter.	Epigenetic silencing of FLC	Flowering	[142]
COLDAIR	The first intron of FLOWERING LOCUS C *FLC, Intronic lncRNA*	Arabidopsis	Interacts with CURLY/PRC2 and localized to chromatin of FLC promoter	Associates with CURLY/PRC2 to mediate silencing of the floral FLC in the winter cold	Repressor of transcription	Flowering	[141]
ASL	Antisense of FLC	Arabidopsis	ASL associates with H3K27me3 regions of FLC	The AtRRP6L proteins function as the main regulators of ASL synthesis	It could function in the maintenance of H3K27 trimethylation	Flowering	[200]
COLDWRAP	Promoter of *FLC,* sense lncRNA	Arabidopsis	5′ half of COLDWRAP physically interacts with CURLY LEAF (CLF), plant homolog of PRC2	COLDWRAP coordinates with a histone H3K27 methyltransferase complex, PRC2, to mediate vernalization-mediated silencing of FLC through the formation of intragenic gene loop within the FLC locus.	Repression of transcription	Flowering	[17]
RIFLA	The first intron of the OsMADS56 gene, Intronic lncRNA	*O. sativa* L (rice)		RIFLA-overexpressing transgenic rice plants flowered earlier than the wild-type	RIFLA formed a complex with OsiEZ1; a homolog of Arabidopsis is required for the inhibition of	Flowering	[20]

					OsMADS56 expression		
FLAIL (flowering-associated intergenic lncRNA) (FLAIL)	Intergenic lncRNA	Arabidopsis	FLAIL acts as a trans-acting RNA molecule		Alternative splicing	Flowering	[201]
LDMAR	pms3 Locus (Intergenic), sense lncRNA	Hybrid rice	-	The locus *pms3* codes for three transcripts; transcript 1 was designated as LDMAR. A variant in the transcript alters the methylation of the locus and the expression	Mutation in the gene causes changes in LDMAR's structure, reduced expression, and cell death	Pollen development, Photoperiod-sensitive male sterility,	[21]
GARR2	Gypsy LTR retrotransposon	*Z. mays* (maize)	*ZmUPL1 during* gibberellin (GA) response	Knockout *GARR2* leads to increases in bud height, second leaf sheath length, and endogenous GA3 levels	Modulates the GA response	Development of plant height	[202]
XR_001593099.1, and 10 lncRNAs*		*Arachis hypogaea* L.(peanut)	LncRNAs target their neighbouring genes (cis-acting)	Co-expression of protein-coding genes of the lnCRNAs and enrichment analysis revealed diverse biological processes and pathways were associated with the lncRNAs	Glycolysis/gluconeogenesis, flavonoid biosynthesis, and galactose metabolism pathways together with others	Peanut seed development	[203]

MSTRG.26204.1		*Beta vulgaris* (sugar beet)	*Bv8_189980_mizi.t1*	The lncRNA co-expressed with vernalization genes, *VRN1*, *VRN1-like*, *VAL1* and *VAL2*		Vernalization	[204]
TWISTED LEAF (TL)	Antisens of R2R3 MYB transcription factor gene locus, OsMYB60	*O. sativa* (rice)	OsMYB60	TL and OsMYB60	TL transcription suppressed the sense gene (OsMYB60) expression by mediating chromatin modifications	Leaf development	
ASCO		Arabidopsis	Interacts with AtNSR (Nuclear speckle RNA-binding protein (NSR) from Arabidopsis)	ASCO decoys (sequester) NSR (RNA binding protein) and prevents NSRs' action on splicing	mRNA Splicing, *ASCO-RNA* expression is upregulated in the *nsra/nsrb* double mutant, which suggests that NSRs not only affect the patterns of alternatively spliced mRNA isoforms but also regulate, directly or indirectly, the expression of lncRNAs	Plant root development	[112]
**Abiotic stress**							
PILNCR1		*Z. mays* (Maize)	*MIR399b*	Phosphate (Pi)-deficiency-induced long-noncoding RNA1 (*PILNCR1*) inhibits		Tolerance to low Pi in maize.	[205]

				ZmmiR399-guided cleavage of *ZmPHO2*			
MSTRG.35921.1		*Glycine max* (Soya beans)	miR166m	miR166m targets different genes related to salinity stress (chloroplastic beta-amylase 1 targeted by miR166m-5p and calcium-dependent protein kinase 1 targeted by miR166m-3p)		Salinity stress	[206]
lncRNA77580		*G. max* (Soya beans)		Overexpressing *lncRNA77580* in soybean could increase the drought tolerance and seed yield by increasing the number of seeds per plant		Drought and Salinity Stress Responses	[207]
IPS1	Intergenic lncRNA	Arabidopsis	Phosphate starvation-induced miR-399	IPS1 RNA sequesters miR-399; PHO2 mRNA is a target of miR-399. IPS1 overexpression results in increased accumulation of the miR-399 target PHO2 mRNA	Phosphate homeostasis	Phosphate signaling	[133]
MSTRG.139242.1		*Camellia sinensis (tea)*	*TEA027212.1* (*Ca^{2+}-ATPase 13*) in the response of tea plants to high salinity	The target gene of the lncRNA *MSTRG.139242.1 co-expressed with the lncRNA and localized near the lncRNA*		Salt stress	[208]
lncRNA354		Gossypium (cotton)	*Acts as competing endogenous RNA miR160b. The miR160b targets GhARF17/18*	Control the expression of the salt stress response-related genes		Salt stress response	[209]
SVALKA	Natural antisense to CBF1, CBF2	Arabidopsis	The longer isoform SVK-L interacts with CBF1 mRNA, the	Interact with CBF2 mRNA to suppress the expression, antisense action on CBF1,	Repress the expression of	Cold response	[143]

	and CBF3/DRE B1A (chr4) cluster, antisense lncRNA		shorter isoform SVK-S may terminate the transcription of CBF1 or interact with the coding region of CBF3 and silence the expression by recruiting PRC2/CURLY LEAF (CLF) to the coding region of the CBF3.	and by binding to the promoter of CBF3 and recruiting	CBF1 and CBF3, interact with gene body, recruit PRC2/CURLY LEAF (CLF to suppress the expression of CBF3		
**Metabolism**							
lncRNA43234		*G. max* (Soya beans)	miRNA10420 that targets XM_014775781.1	lncRNA43234-miRNA10420-XM_014775781.1 network related to lipid synthesis was screened out by full-length transcriptome sequencing for Wild type (WT) soybean. Overexpression of *lncRNA43234* resulted in increased protein content and decreased oleic acid content in *Arabidopsis thaliana* seeds	Involves in fatty acid transport, lipid synthesis, and cell division	Oil metabolism in soybean	210]
MSTRG.22563		*Brassica napus* L (Rapeseed)	Decreased expression of genes related to lipid metabolism and the metabolites related to respiration and TCA cycle	The seed oil content decreases by 3.1%–3.9% following the overexpression of *MSTRG.22563*	Lipid metabolism, respiration, and TCA cycle	Oil metabolism in Rapeseed	

MSTRG.86004		*B. napus* L (Rapeseed)	Increased expression of genes involved in fatty acid synthesis like LEC1, but decreased expression of genes related to triacylglycerol (TAG) synthesis in TMSTRG.86004 transgenic seeds.	The seed oil content increases by approximately 2% if *MSTRG.86004* is overexpressed	Seed embryonic development, fatty acid synthesis	Oil metabolism in Rapeseed	[211]
LTCONS_0005400 3 (another 9 lncRNA)#		*C. sinensis (tea)*	Targets 4CL based on co-expression	Altered expression of the lncRNAs and their targets in solar-withered leaves #		Flavonoid metabolism	[212]
LTCONS_0009314 0 (other lncRNA)$		*C. sinensis (tea)*	Targets DXS	Altered expression of the lncRNAs and their targets in solar-withered leaves $		Terpenoid metabolic pathway	[212]
*MSTRG 2723.1 (three other lncRNA)***		Gossypium (cotton)		LancRNA mediates the expression of key genes related to fatty acid metabolism, the MYB25-mediated pathway, and pectin metabolism to regulate fiber synthesis	Fatty acid metabolism	Fiber development by changing Fatty acid metabolism	[213]
**Others**							
LTCONS_0004066 7 (other lncRNAs)$		*C. sinensis (tea)*	Targets LOX	Altered expression of the lncRNAs and their targets in solar-withered leaves $		JA/MeJA biosynthesis and signal transduction pathway	[212]

lncRNA00022274	An antisense overlap with the MYB gene, BntWG10016451, Antisense lncRNA	*Boehmeria nivea* L. Gaud (ramie plant)	MYB gene (*BntWG10016451*	lncR00022274 exhibited downregulated expression in barks with growing fibers.		Fiber production	[214]
lncRNA000170	Transcribed from the complementary strand of *Solyc10g006360*	*Solanum lycopersicum* L. (Tomato)	LncRNA000170 induces expression of Solyc10g006360	Affect multicellular trichome formation by inducing target gene expression. Several trichome regulators, such as Wo, H, SlCycB2, and SlCycB3, are downregulated by lncRNA000170 overexpression	Trichome regulators	Plant defense	[215]
ELENA1	Intergenic	*Arabidopsis thaliana*	ELENA1 directly interacts with Mediator subunit 19a (MED19a) and affects enrichment of MED19a on the *PR1* promoter.	as a factor enhancing resistance against *Pseudomonas syringe* pv *tomato* DC3000	ELENA1 knockdown show decreased expression of *PATHOGENESIS-RELATED GENE1* (*PR1*)		[216]

Abbreviations

FLC- FLOWERING LOCUS C; COLDWRAP- Cold of winter-induced noncoding RNA from the promoter; COLDAIR- Cold Assisted Intronic noncoding RNA; APOLO- AUXIN-REGULATED PROMOTER LOOP; LDMAR- Long-day-specific male-fertility-associated RNA; ASCO-Alternate splicing competitor long noncoding RNA; IPS1- INDUCED BY PHOSPHATE STARVATION 1; HID1- HIDDEN TREASURE 1; ASL- ANTISENSE LONG; PILNCR1- Pi-deficiency-

induced long-noncoding RNA1; GARRs- GIBBERELLIN-RESPONSIVE lncRNAs; TRABA- Trans acting of BGLU24 by lncRNA; ELENA1- ELF18-INDUCED LONG-NONCODING RNA1

*XR_001593099.1, MSTRG.18462.1, MSTRG.34915.1, MSTRG.41848.1, MSTRG.22884.1, MSTRG.12404.1, MSTRG.26719.1, MSTRG.35761.1, MSTRG.20033.1, MSTRG.13500.1, and MSTRG.9304.1

#**LncRNAs involved in flavonoid metabolism in tea:**
LTCONS_00054003 (targeting 4CL), LTCONS_00060939 (targeting CHI), LTCONS_00056216 (targeting F3H), LTCONS_00044497 (targeting F3′H), LTCONS_00031811 (targeting FLS), LTCONS_00001863 (targeting CCR), LTCONS_00000233 (targeting CAD), LTCONS_00090121 (targeting CAD), LTCONS_00030131 (targeting HCT), and LTCONS_00101116 (targeting HCT).

$LncRNAs involved in the terpenoid metabolic pathway and the JA/MeJA biosynthesis and signal transduction pathway in tea:
*LTCONS_00093140 (targeting DXS), LTCONS_00012676 (targeting CMK), LTCONS_00092790 (targeting PMK), LTCONS_00002173 (targeting HDS), LTCONS_00078708 (targeting HDR), LTCONS_00039845 (targeting GGPPS), LTCONS_00025739 (targeting AACT), LTCONS_00091745 (targeting MVK), LTCONS_00043160 (targeting TPS) (***terpenoid metabolic pathway)** , LTCONS_00040667 (targeting LOX), LTCONS_00087608 (targeting AOS), LTCONS_00035664 (targeting AOC), LTCONS_00032547 (targeting OPR), LTCONS_00064473 (targeting ACX), LTCONS_00087182 (targeting ACX), and LTCONS_00061187 (targeting MFP2) (**JA/MeJA biosynthesis and signal transduction)**
** MSTRG 3390.1, MSTRG 48719.1, and MSTRG 31176.1 also showed potentially important roles in fiber development, and the co-expression analysis between lncRNAs and targets showed the distinct models of different lncRNAs and complicated interactions between lncRNAs in fiber development of cotton [213].
