# Supplementary material for: LncRNAOmics: A Comprehensive Review of Long Non-Coding RNAs in Plants
Source: Genes (Basel). 2025 Jun 29;16(7):765. doi: 10.3390/genes16070765 (PMC12294436; doi:10.3390/genes16070765)
Supplement: Supplementary file 1 [file genes-16-00765-s001.zip › Supplementary Table S2.pdf]

Supplementary Table S2: Plant disease-related lncRNAs

Disease	Description	Plant	lncRNA/Involvement	Reference
Downy mildew	Caused by water molds, and affects fruits, vegetables, and flowers	*Brassica rapa* L. ssp. Pekinensis (Chinese cabbage)	MSTRG.19915 (antisense to BrMAPK15) Resistance to Downy mildew	[217]
Ligon-lintless-1 mutant	Defects in cotton fibre development	*Gossypium hirsutum* (Cotton)	LNC_017085 Down-regulated in the mutant line	[218]
Pathogen induced genes	Knockdown of ASCO induces pathogen related genes	Arabidopsis	ASCO knocked-down induces pathogen-related genes	[117]
Shade avoidance syndrome (SAS)	Occurs in plants experiencing vegetative shade, triggering a series of morphological and physiological changes for the plants to reach more light	Arabidopsis	LncRNA PUAR physically associates with TF PIF7 and represses the shade-mediated induction of phytochrome A (PHYA)	[219]
Respond to nitrate and nitrate regulation.	Nitrogen availability modulates defence against various infections [220-222]	Arabidopsis	The lncRNA T5120 regulates nitrate response and assimilation and may modifies defence against infections	[223]
Infection with *Xanthomonas oryzae* pv Oryzae	Bacterial infection causes a serious blight of rice, other grasses, and sedges	*Oryza sativa*	Overexpression of ALEX1 enhances the resistance to Xoo and activates Jasmonate (JA) signaling	[168]
Sugarcane mosaic virus (SCMV)	Affects photosynthesis and growth of sugarcane, leading to a significant decrease in cane yield and sucrose content	Sugarcane	*Silencing lncRNA10865* aggravated SCMV symptoms. *Silencing lncRNA14234* alleviated SCMV symptoms	[166]
Clubroot disease	A soil-borne disease caused by the pathogen Plasmodiophora brassicae, causing root	*Brassica napus* L (Rapeseed)	Hundreds of differentially expressed lncRNAs were identified in the roots of resistant plants challenged with	[224]

	swellings and stunted growth, potentially leading to significant yield losses.		*Plasmodiophora brassicae*. The most of the genes targeted by these lncRNAs associated with plant–pathogen interactions and hormone signaling pathways	
Tea leaf spot disease	Often caused by fungi like Didymella segeticola or Exobasidium vexans	*Camellia sinensis (tea)*	*MSTRG.20036*, *MSTRG.3843*, *MSTRG.26132*, and *MSTRG.56701* through cis-regulatory mechanisms	[225]
