# Supplementary material for: LncRNAOmics: A Comprehensive Review of Long Non-Coding RNAs in Plants
Source: Genes (Basel). 2025 Jun 29;16(7):765. doi: 10.3390/genes16070765 (PMC12294436; doi:10.3390/genes16070765)
Supplement: Supplementary file 1 [file genes-16-00765-s001.zip › Supplementary Text S1 and S2 .pdf]

Supplementary Text S1: Biogenesis and mechanism (s) of actions of microRNA

(Related to section 2.1 in the main text)

2.1 Biogenesis and mechanism (s) of actions of microRNA

Intergenic microRNA genes are typically transcribed by DNA-dependent RNA polymerase II (RNA pol II) using their own separate promoters. In contrast, intronic microRNA genes are co-transcribed by the same RNA pol II from shared promoters alongside their corresponding protein-coding genes, resulting in the production of primary microRNAs (pri-miRNAs). Similar to protein-coding genes, microRNA genes are regulated by chromatin remodeling, transcription factors, and various epigenetic processes. Modified primary microRNAs, or pri-miRNAs, feature a 3′ poly(A) tail and a 5′ methylguanosine (m^7G) cap. Their distinctive hairpin structure consists of a lower stem, a miRNA/miRNA* duplex, an upper stem, and a terminal loop. Dicer-like RNase III endonucleases (DCLs) are particularly adept at recognizing and digesting specific stem-loop combinations. Different plant species have varying numbers of Dicer-like (DCL) proteins, with DCL1 primarily responsible for processing microRNAs (miRNAs). This function is aided by the zinc-finger protein Serrate (SE) and the double-stranded RNA-binding protein Hyponastic Leaves 1 (HYL1). However, other DCL proteins can also play a role in miRNA biogenesis. For instance, DCL4 synthesizes miR822 and miR839 in *Arabidopsis thaliana*, while OsDCL3a produces a specific class of 24-nucleotide miRNAs in rice. The protein Hasty (HST), a plant equivalent of the animal protein Exportin 5 (EXPO5), facilitates the export of methylated miRNA-miRNA* duplexes from the nucleus to the cytoplasm. Once in the cytoplasm, the duplexes are loaded onto AGO1 and incorporated into the RNA-induced silencing complex (RISC). Evidence shows that EXPO1 assembles the RISC within the nucleus and then exports it to the cytoplasm. However, current findings do not dismiss the possibility that certain miRNAs are formed in the cytoplasm and exported in their duplex forms, at least during biogenesis. The AGO1 protein, the primary effector among the ten AGO proteins identified in Arabidopsis, is formed through the selective assembly of one strand from a miRNA/miRNA∗ duplex, known as the guide strand or miRNA. The other strand, the passenger strand or miRNA∗, is expelled and subsequently broken down [178-180]. The biogenesis of miRNA is shown in **Supplementary Text Figure STF1.**

In contrast to animals, where microRNAs typically bind to the 3'-untranslated regions (UTRs) of mRNA, the guide strand in the RISC-AGO1 complex interacts with mRNA through sequence complementarity, mainly within the coding regions of the mRNA. In plants, however, miRNAs may also occasionally interact with the 3'- or 5'-untranslated regions [181,182].

Plant microRNAs and their target messenger RNAs (mRNAs) exhibit almost perfect matching. It was previously believed that mRNA cleavage was the primary mechanism by which plant miRNAs function. The high degree of sequence complementarity between miRNAs and mRNAs typically leads to mRNA cleavage. However, there have also been reports of translation inhibition resulting from interactions between miRNAs and mRNAs [183]. MicroRNAs play a crucial role in various biological processes by negatively regulating the expression of protein-coding genes. Several factors control their expression at the transcriptional level, including histone modifications, transcription factors, and DNA methylation. Additionally, the conversion of primary transcripts into mature microRNAs and the stability of these microRNAs can be influenced by the levels of mature microRNA present [179].

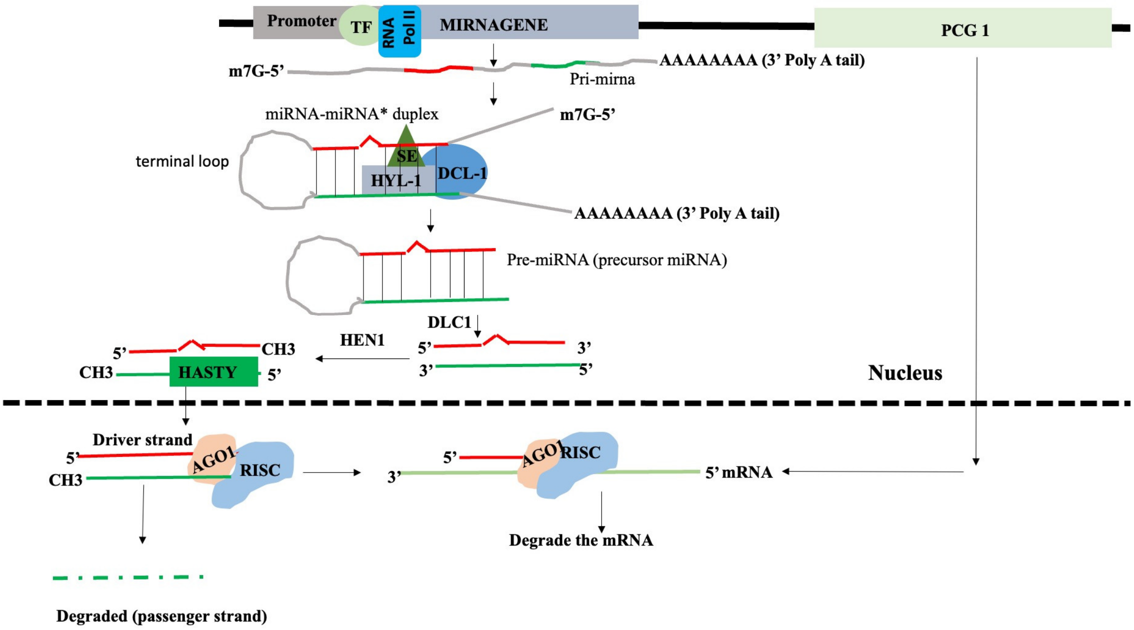

Supplementary Text Figure STF1 Simplified steps for the biogenesis and actions of microRNA in plants

Supplementary Text S2 : Long Noncoding RNAs in response to abiotic stress

(Related to section 5.1 in the main text)

5.1 Long Noncoding RNAs in response to abiotic stress

Long non-coding RNAs (lncRNAs) play a crucial role in helping plants adapt to abiotic stressors such as drought, salinity, and temperature fluctuations by regulating gene expression and inducing epigenetic changes. In response to abiotic stress, plants can produce thousands of

lncRNAs, which can be identified using next-generation sequencing technologies. These lncRNAs demonstrate dynamic expression patterns in response to various environmental cues. While our understanding of the roles of lncRNAs in managing abiotic stress is still evolving, there are currently several reviews available that examine their functions in this context [184,185].

5.1.1 Drought

In Arabidopsis, the long non-coding RNA, DROUGHT INDUCED lncRNA (DRIR), is primarily expressed at low levels in the root, inflorescence, embryo, shoot, and leaf nuclei under normal conditions, without stress. However, its expression significantly increases in response to drought, salt stress, and treatments with abscisic acid (ABA). Transgenic plants that overexpress the lncRNA DRIR show enhanced resistance to salt stress and drought. DRIR contributes positively by reducing water loss through transpiration and improving drought resistance. Transcriptome analysis of the DRIR overexpressing lines and the drirD mutant line revealed significant changes in the expression of genes related to ABA signaling, water transport, and various stress-relief mechanisms [154]. A transcriptome analysis of two Tibetan wild barley varieties with different levels of drought tolerance revealed 535 long non-coding RNAs that are differently expressed under drought stress. Functional enrichment analysis and the construction of lncRNA-mRNA relationships, both in cis and trans, indicated that ascorbate/aldarate metabolism, kinase signaling, and plant hormone signal transduction were significantly enriched with the expected targets of these lncRNAs. It was suggested that the drought-tolerant cultivar may be supported by SMG1, a serine/threonine protein kinase, which is predicted to be targeted by several of the lncRNAs [155]. A genome-wide transcriptional study identified 1,535 lncRNAs that respond to drought stress in maize at various developmental stages. These drought-responsive lncRNAs showed greater selectivity for specific tissues and developmental stages than protein-coding genes. Notably, more lncRNAs exhibited changes in expression during the reproductive stage R1, indicating that this stage is the most vulnerable to drought stress. The study also identified 653 potential lncRNA-messenger RNA (mRNA) pairings based on the projected mRNA targets of the deregulated lncRNAs. Of these pairings, 124 function in a cis-acting manner while 529 operate in trans. For instance, it was predicted that the neighboring lncRNA MSTRG.6838.1 would target the gene vpp4, which encodes a vacuolar (H+)-pumping ATPase component. Under drought stress, both vpp4 and MSTRG.6838.1 were found to be downregulated. The expressions of vpp4 and MSTRG.6838.1 were correlated significantly in different tissues and

development stages. Enrichment analysis indicated that the predicted targets were significantly enriched in molecular functions associated with oxidoreductase activity, water binding, and electron carrier activity [186]. A transcriptome study of drought-tolerant and drought-sensitive cultivars in response to water deficit stress has been conducted in rapeseed (*Brassica napus* L.). In drought-sensitive plants, 449 down-regulated and 257 up-regulated lncRNAs were identified, while drought-tolerant plants displayed 369 down-regulated and 108 up-regulated lncRNAs. The co-expression networks for drought-tolerant plants included 145 network nodes and 5,175 connections according to lncRNA-mRNA interaction network analysis, whereas drought-sensitive co-expression networks had 305 network nodes and 22,327 connections. Furthermore, in drought-tolerant plants, 34 transcription factors (TFs) were found to correlate with 126 differentially expressed lncRNAs, while in drought-sensitive plants, 45 TFs correlated with 359 differentially expressed lncRNAs. Differential expression analysis of lncRNAs revealed that up- and down-regulated mRNAs co-expressed with lncRNAs involved different metabolic pathways and regulatory mechanisms across the two genotypes. Some lncRNAs showed co-expression with BnaC07g44670D, which is associated with plant hormone signal transduction. Additionally, several mRNAs near XLOC_052298, XLOC_094954, and XLOC_012868 were associated with signal transport and defense/stress response [187]. A total of 40 lncRNAs, 23 miRNAs, and 103 mRNAs that are differentially expressed in rice were combined to create a drought-responsive lncRNA-miRNA-mRNA network, also known as a ceRNA network. It was discovered that lncRNA TCONS_00021861 could regulate YUCCA7 by sponging miR528-3p, confirming its potential role as a ceRNA. This interaction activates the indole-3-acetic acid (IAA) biosynthesis pathway, which contributes to the development of drought stress tolerance [188]. In rice under drought stress, 191 lncRNAs, 2115 mRNAs, and 32 miRNAs were found to be differentially expressed. It is projected that these differentially expressed lncRNAs will target thousands of trans-target genes as well as 3,284 adjacent protein-coding genes (100 kb upstream and downstream). Protein synthesis, fatty acid degradation, photosynthesis, arginine and proline metabolism, carbon metabolism, cysteine, and methionine metabolism, porphyrin and chlorophyll metabolism, hormone signal transduction, and other metabolic pathways are all significantly impacted by differentially expressed genes, including lncRNAs and their targets, according to functional enrichment analysis. Sixteen drought-specific lncRNAs are expected to target the drought-responsive gene Os05g0586700, which plays a vital role in plant membrane repair during drought. It has been suggested that MSTRG.28732.3 responds to dryness by controlling the pathway involved in chlorophyll production [189].

5.1.2 Salt/saline stress

The adverse effects of excessive salt concentrations in soil and/or water, especially sodium (Na+) and chloride (Cl-) ions, on plant growth and development are referred to as salt stress. Reduced agricultural yields are the result of osmotic and ionic imbalances, as well as decreased water intake caused by salt stress. Salt stress modifies the transcription of several genes, including lncRNAs, just like other abiotic stressors do. Salt (NaCl) treatment in Arabidopsis resulted in increased expression of npc536, npc60, and npc82 in the roots and leaves; these genes were thought to be salt-responsive lncRNAs. Under salt stress, npc536 overexpression promotes root growth, most likely by affecting the translation of CONSERVED OLIGOMERIC GOLGI COMPLEX 5 (AtCOG5) mRNA [190]. Arabidopsis lncRNA DRIR contributes to drought resistance. Additionally, DRIR provides tolerance to salinity stress and is likewise salt-stress inducible. Stress-related genes P5CS1, RD29A, RD29B, and NAC3 were more highly expressed in salt-treated mutant drirD plants and DRIR overexpressing seedlings. Genes linked to the ABA signaling pathway showed enhanced expression under drought conditions, while no genes linked to ABA signaling were found under salt stress. This finding suggests that under particular stress situations, DRIR controls stress-related genes via distinct methods [154]. In cotton, the expression of lncRNA973 is increased in response to salt stress. The ability of the lncRNA to tolerate salinity stress could be due to its ability to regulate miR399 directly and the expression of GhPHO2 indirectly, as GhPHO2 is a validated target of miR399. Knocking out lncRNA973 in cotton seedling roots significantly increased miR399 expression [191]. The aquatic plant duckweed *Spirodela polyrhiza* was treated with 100 mM NaCl to investigate the lncRNAs implicated in the response to salt stress. With this treatment, the relative growth rate was reduced by around 60% 96 hours after the treatment began. At 0, 6, 12, and 24 hours following the start of treatment, RNA samples were taken. 2,185 lncRNAs were found using the aquatic plant duckweed *Spirodela polyrhiza*, that was treated with high concentrations of NaCl (100 mM) at various intervals. Of these lncRNAs, 2,269 were identified as intergenic, and 566 were naturally occurring antisense lncRNAs. A total of 185 lncRNA were differentially expressed. In addition, 38, 32, and 25 lncRNAs were expressed exclusively after 6, 12, and 24 h. To characterize the functions of salt-responsive lncRNAs that regulate nearby protein-coding genes (cis-regulation) between 10 kb upstream and 100 kb downstream of lncRNAs were selected. A total of 42 lncRNA-mRNA pairs were identified. The protein-coding genes were involved in cell walls, cell cycle, carbon metabolism, ROS regulation, hormone metabolism, and transcription factor. Similarly, trans acting regulation was determined from the co-

expression of protein-coding genes with the lncRNAs (trans regulation by the lncRNAs). According to the co-expressed protein-coding genes, the lncRNAs have the following functions: transport, RNA transcription, secondary metabolism, hormone metabolism, amino acid metabolism, cell and cell wall metabolism, and photosynthesis. Therefore, the lncRNAs that are differently expressed in response to cold stress are probably implicated in pertinent cold-responsive processes [150].

A tolerant genotype (FL478) and a sensitive genotype (IR29) were used in an experiment to investigate the role of long non-coding RNAs (lncRNAs) in rice during salt stress. After being placed in Yoshida solution containing 150 mM NaCl for 24 hours, 21-day-old seedlings had their RNA extracted and sequenced. A total of 15,131 lncRNA sequences were identified in FL478 and 16,256 in IR29. These sequences included intergenic, intronic, antisense, and sense types. In plant roots of two resistant (ICCV 10, JG 11) and two susceptible (DCP 92-3, Pusa 256) types treated with varying doses of NaCl up to a maximum of 150 mM NaCl, the function of lncRNA in chickpeas' response to salt stress was investigated. 4446 differently expressed lncRNAs were found under different salt treatments when comparing salt-tolerant and salt-sensitive cultivars. Ten kb upstream and downstream of the lncRNA loci were searched to find genes that are cis-actingly controlled by lncRNAs. Each salt-tolerant line was contrasted with the two salt-sensitive lines to ascertain the function of lncRNAs in the salt stress response. It was predicted that 3373 lncRNAs were regulating their target genes in cis regulating manner, and 80 unique lncRNAs were identified as interacting partners of 136 different microRNAs. The differentially expressed lncRNAs were shown to control in cis genes related to the response to saline stress like potassium transporter, transporter family genes, serine/threonine-protein kinase, aquaporins like TIP1-2, PIP2-5 and transcription factors like, AP2, NAC, bZIP, ERF, MYB, and WRKY. Result suggests that lncRNA can regulate the response to salt by controlling gene expression by acting in cis or as an endogenous target mimic [152]. Analysis of differential expression revealed nine lncRNAs in IR29 and four in FL478. Among these, LncRNA.2-FL was particularly notable in the tolerant genotype, as it regulates 172 mRNAs in trans and a gene that codes for a pentatricopeptide repeat protein in cis. Overall, LncRNA.2-FL plays a crucial role in promoting the growth of lateral roots by rerouting auxin to help mitigate high salt concentrations [192]. Two lncRNAs (lncRNA.2-FL, lncRNA.3-FL) were identified in response to salt in rice (FL478, salt tolerant). Altered expression of lncRNAs like lncRNA.2-IR, lncRNA.3-IR, lncRNA.5-IR, and others was also identified in parental IR29 (salt susceptible), indicating these lncRNAs are likely to be involved in salt stress response. Altered expression of many lncRNAs was identified in osmotic stress-treated leaf and root samples from *Medicago truncatula*. LncRNAs TCONS_00048391 and TCONS_00010856 from Chinese Cabbage (*Brassica rapa* sp. Chinensis) were associated with heat stress; the lncRNA CIL1 from Arabidopsis was associated with cold stress [28].

5.1.3 Cold

A total of 318 lncRNAs that respond to cold and/or drought stress have been identified in cassava, a starchy root vegetable native to South America. These lncRNAs typically co-express with their neighboring genes in either a concordant or discordant manner. Many lncRNAs have been associated with the formation of secondary metabolites, the transduction of hormone signals, and the metabolism of sucrose, as indicated by studies of the trans-regulatory network. Notably, a cold-repressive lincRNA, termed lincRNA159, has been discovered in cassava. Functional studies show that miR164 targets lincRNA159, which is encoded from the intergenic region. Additionally, it was found that 16 out of 682 lncRNAs function as competing endogenous RNAs (ceRNAs) [193]. The MADS AFFECTING FLOWERING4 (MAF4) gene in Arabidopsis is the source of transcription for the natural antisense lncRNA MAS. The transcriptional level of MAS and its sense counterpart MAF4 mRNA is elevated in response to cold stress. The recruitment of WDR5a to MAF4 is further mediated by subsequent contact of MAS with WDR5a, a fundamental element of the COMPASS-like complexes, which enhances the histone modification H3K4me3, which is a characteristic of activated genes. These findings demonstrate that natural antisense-lncRNAs control gene expression throughout the vernalization response [137]. Assessing the fraction of lncRNAs that respond to abiotic stresses like heat, cold, salt, and drought, 1077 differentially expressed lncRNA transcripts, including 509 lncRNAs having sequence similarity to transposable elements (TEs) (TE-lncRNAs), were identified [153]. In grapes, the expression of 813 lncRNAs (487 increased and 326 decreased) is deregulated in response to cold stress. It is predicted that 203 differentially expressed lncRNAs regulate 326 target genes. Differentially expressed lncRNAs are predicted to target stress response-related genes like CBF and WRKY transcription factors, late embryogenesis abundant (LEAs) genes, and peroxisome biogenesis-related genes [156]. A total of 983 lncRNAs were identified in the leaves, while 1,288 lncRNAs were found in the roots of the model legume Medicago truncatula. These were classified as cold-responsive lncRNAs. It was noted that the leaves and roots exhibited different sets of cold-responsive lncRNAs. Most of these lncRNAs were classified as intronic lncRNAs, with a smaller percentage being natural antisense lncRNAs. Through co-expression and co-localization analyses, it was determined that the target genes of these lncRNAs were primarily associated with defense responses, intracellular protein transport, vesicle-mediated transport, metabolic processes, transcription, protein translation, and glycosylation [159]. In wheat, the lncR9A, lncR117, and lncR616 interact with the miR398 to regulate the expression of COPPER/ZINC SUPEROXIDE DISMUTASE 1

(CSD1) and improve tolerance to cold stress [194]. A total of 316 long lncRNAs were identified as cold-related lncRNAs in cassava. One of these, the cold-responsive intergenic lncRNA 1 (CRIR1), enhances cold stress resistance by interacting with the cold shock domain protein MeCSP, which improves translation efficiency at low temperatures. Functional studies revealed that while cold stress-related marker genes (such as MeCBFs, MeCOR, and MeICE1) were not differentially expressed, other cold stress-related genes (including MeNAC, MeNF-YA, and MeGOLS, along with galactinol synthases) showed elevated expression in both wild-type (WT) and CRIR1-overexpressing plants. This suggests that CRIR1 enhances cold tolerance in plants through a mechanism that does not rely on CBF, possibly involving auxin signaling [146]. In response to cold stresses, altered expression of lncRNAs like COLDWARP, COOLAIR, COLDAIR, COLDWRAP, SVALKA, etc., has been reported. COLDWARP, COOLAIR, COLDAIR, and COLDWRAP have been shown to induce chromatin modification to alter the target gene expression [176,184].

5.1.4 Heat

Heat stress significantly restricts crop production globally and adversely affects plant growth and development. When plants are exposed to high temperatures, they produce genes that encode long non-coding RNAs (lncRNAs), regulatory proteins such as transcription factors (TFs) and protein kinases, as well as enzymes that help scavenge reactive oxygen species (ROS). There have been several documented cases of altered lncRNA expression in response to heat stress. For example, during heat stress in wheat, 77 lncRNAs were identified, with TahlnRNA27 being recognized as a potential precursor for microRNA (miRNA) [157]. The responses of Arabidopsis seedlings to cold, heat, salt, drought, and bright light were found to be represented by 245 DE poly(A)+ and 58 poly(A)- lncRNAs. Several unregulated lncRNAs were shared by several stressors. Only 15 of the unregulated lncRNAs in response to stress were heat-responsive, with lncRNA AT3G43190 being the only one. The findings showed that all lncRNAs have a higher stress specificity than coding genes, with poly(A)− lncRNAs having a higher stress specificity than poly(A)+ lncRNAs [149]. The female gametophyte in Arabidopsis expresses HSFB2a, which controls the development of both the vegetative and gametophyte stages. AsHSFB2a, a natural antisense lncRNA, selectively inhibits the expression of HSFB2a. When exposed to heat stress, the antisense regulation of HSFB2a by elevated asHSFB2a counteracts the repressive effects of HSFB2a, hence promoting growth and continued development [195]. In the Chinese cabbage, the lncRNA TCONS_00016454 might be involved in heat tolerance via Brassinosteroid (BR) signaling pathway as this lncRNA showed antagonistic expression patterns with its target, a BES1/BZR1 homolog in response to high temperatures in this system [196].

Under heat stress, 34 differentially expressed lncRNAs were identified in B. rapa (Chinese cabbage), along with 192 putative target genes. Co-expression networks involving the differentially expressed lncRNAs, mRNAs, and microRNAs suggested a link between phytohormones, such as salicylic acid and brassinosteroid (BR) pathways, and heat tolerance. Notably, 25 lncRNAs were significantly co-expressed with 10 heat-responsive genes. Furthermore, it was observed that 35 miRNAs are expected to have 39 lncRNAs as endogenous target mimics. One specific endogenous target mimic for bra-miR164a is a heat-responsive lncRNA designated as TCONS_00048391. The miR164a targets the gene NAC1 (Bra030820). Therefore, the association of TCONS_00048391 with miR164a may sequester the microRNA, preventing it from interacting with its target NAC1 [196,197]. When RNA from immature leaves of radish (Raphanus sativus L.) were sequenced after being exposed to 40°C for 6 hours, 169 lncRNAs were found to be differently expressed, with 117 lncRNAs showing upregulated expression and 52 showing downregulated expression. Based on the deregulated neighboring protein-coding genes, the functions of the deregulated lncRNAs were anticipated. The pathways involved in hormone signal transduction, oxidative phosphorylation, peroxisomes, carbon fixation, and photosynthesis were all enhanced. The metabolic processes of energy production, carbon metabolism, and amino acid biosynthesis have also been linked to lncRNAs. An elevated lncRNA increased a heat shock protein's mRNA [158]. There are 204 high-temperature-responsive lncRNAs in a species of poplar (Populus simonii) that can withstand abiotic stress. These lncRNAs were expected to target 119 trans and 100 cis genes. The target genes were more abundant in signaling pathways, anatomical structure development, and stress response. TCONS_00202587 obstructs transcription by attaching itself to upstream regions of its target gene. lncRNA TCONS_00260893 increases the calcium influx at elevated temperatures. In model plants Arabidopsis under heat stress, expression of these two lncRNA targets reduced DNA damage, prevented membrane peroxidation, and enhanced photosynthetic protection and recovery [198]. In heat-resistant elite maize inbred line, 993 heat-responsive differentially expressed lncRNAs were identified under heat stress. These lncRNAs are predicted to target thousands of nearby genes and co-express with the lncRNAs. Several relevant important biological processes and pathways involved in heat response like stress response, hormone signaling, metabolism, photosynthesis, and spliceosome were highly enriched with the target genes of the lncRNAs [151]. One possible explanation for the deregulated lncRNAs seen in various plants in response to stress in general and heat stress in particular is alterations in DNA methylation. In *Populus simonii* (poplar), stress-specific differentially methylated regions (SDMRs) comprising 17 lncRNA genes and 16 microRNA genes have been discovered. The sense strand in the differentially methylated region SDMR162 exhibited greater amounts of methylation of CG, CHG, and CHH contexts than the antisense strand during osmotic and cold stress. On the other hand, in CG, CHG, and CHH contexts, the antisense strand showed noticeably more methylation than

the sense strand under heat and salt stress. De novo methylation and demethylation occurred simultaneously, and the amounts of methylation on both strands gradually rose. Among the abiotic stresses tested, methylation levels rose most rapidly under heat stress. The differentially methylated region SDMR162 encompassed lncRNA PsiLNCRNA00268512/TCONS_00268512 and the miR396e gene. PsiLNCRNA00268512 is up-regulated by osmotic and cold stress initially and decreased after 12h [95].

5.1.5 Others

Cadmium (Cd) stress induces 69 long non-coding RNAs (lncRNAs) and represses 75 lncRNAs in rice. Among these, 120 are classified as intergenic, 23 as antisense, and 1 as intronic. Based on the proximity of protein-coding genes to the lncRNAs (cis regulation), 120 of the differentially expressed lncRNAs interacted with 362 differentially expressed protein-coding genes, resulting in 386 lncRNA-mRNA interactions. The genes regulated in cis were associated with pathways related to secondary metabolism, sulfur metabolism, amino acid metabolism, carotenoid production, and photosynthesis. The co-expressed protein-coding genes helped identify those under trans regulation. Additionally, secondary metabolites, including phenylpropanoids and phenylalanine, were linked to trans-regulated genes, and Cd stress significantly altered genes involved in the photosynthetic system. All of these results indicate that lncRNAs may regulate genes of cysteine-rich peptide metabolism in cis, as well as secondary metabolites and photosynthesis in trans, to activate various physiological and biochemical reactions to respond to excessive Cd [147]. In barley, lncRNAs that are responsive to boron (B) are identified in the roots and leaves. Compared to the root, leaves show a higher number of B-responsive differentially expressed lncRNAs. Only cultivars that are B-tolerant exhibit over-expression of lncRNAs such as TCONS_00045190 and TCONS_00056415 in response to excessive B treatment. Functional analysis of the coding transcripts co-expressed with lncRNAs showed that only in the leaf were molecular functions such as ion transport, localization establishment, and stimulus response considerably enriched [134]. Potassium (K) is essential for plant growth and determines crop yield, quality, and especially leaf development in tobacco. Analysis of transcriptome from tobacco treated with 2 mM of potassium (control) and 0.01 mM of potassium (low potassium condition) revealed that 242 lncRNAs were differentially expressed in low potassium conditions. Co-expression network construction revealed 78 lncRNA-mRNA potential regulation modules. Further module-trait analysis and module membership ranking nine modules, including 616 mRNAs and 146 lncRNAs, showed a high correlation with K treatments, and 20 hub

potassium-responsive lncRNAs were predicted. Gene ontology (GO) analysis showed potassium starvation inducing the anti-oxidative stress pathway, which is consistent with the physiological results. LncRNAs, like MSTRG.6626.1, MSTRG.11330.1, and MSTRG.16041.1 were correlated with a bench of MYB, C3H, and NFYC transcript factors in response to the stress. The result shows that lncRNAs respond to K concentration from starvation and sufficient supply [148]. Phosphate (Pi)-deficiency-induced long-noncoding RNA1 (*PILNCR1) i*nhibits ZmmiR399-guided cleavage of *ZmPHO2,* affecting the ability of maize to tolerate low-Pi conditions. Knockout *GARR2* in maize leads to increases in bud height, second leaf sheath length, and endogenous gibberellin GA3 levels. Interaction of lnc663 in bread wheat (*Triticum aestivum* L.) with miR1128 has been shown to regulate PDAT-like gene, involved in fat biosynthesis [119].
